# Fluorescent Probes as a Tool in Diagnostic and Drug Delivery Systems

**DOI:** 10.3390/ph16030381

**Published:** 2023-03-01

**Authors:** Nikolai I. Georgiev, Ventsislav V. Bakov, Kameliya K. Anichina, Vladimir B. Bojinov

**Affiliations:** 1 Department of Organic Synthesis, University of Chemical Technology and Metallurgy, 8 Kliment Ohridsky Str., 1756 Sofia, Bulgaria; 2 Bulgarian Academy of Sciences, 1040 Sofia, Bulgaria

**Keywords:** fluorescent probes, drug delivery, cell imaging, fluorescent diagnostic and therapy, molecular logic gates, fluorescent–drug conjugates, theranostics

## Abstract

Over the last few years, the development of fluorescent probes has received considerable attention. Fluorescence signaling allows noninvasive and harmless real-time imaging with great spectral resolution in living objects, which is extremely useful for modern biomedical applications. This review presents the basic photophysical principles and strategies for the rational design of fluorescent probes as visualization agents in medical diagnosis and drug delivery systems. Common photophysical phenomena, such as Intramolecular Charge Transfer (ICT), Twisted Intramolecular Charge Transfer (TICT), Photoinduced Electron Transfer (PET), Excited-State Intramolecular Proton Transfer (ESIPT), Fluorescent Resonance Energy Transfer (FRET), and Aggregation-Induced Emission (AIE), are described as platforms for fluorescence sensing and imaging in vivo and in vitro. The presented examples are focused on the visualization of pH, biologically important cations and anions, reactive oxygen species (ROS), viscosity, biomolecules, and enzymes that find application for diagnostic purposes. The general strategies regarding fluorescence probes as molecular logic devices and fluorescence–drug conjugates for theranostic and drug delivery systems are discussed. This work could be of help for researchers working in the field of fluorescence sensing compounds, molecular logic gates, and drug delivery.

## 1. Introduction

Due to their importance in chemical laboratories, healthcare, industry, food production, biotechnological processes, and environmental and life sciences, a great deal of attention is currently being paid to the design and synthesis of new materials with chemosensing properties [1,2,3,4,5,6]. The recent progress in this field has resulted in a large number of different architectures. Among them, fluorescent probes have been the subject of major research interest [7,8,9,10,11,12,13,14,15,16]. Mainly, their attractiveness lies in their fluorescence signaling output, which provides a rapid response time, high efficiency and sensitivity, great spatial resolution, and cheap and affordable equipment suitable for field analysis. Moreover, owing to their smaller size, together with their safe and indestructible signaling nature, they are excellent tools in diagnostic medicine and real-time bioimaging [17,18,19,20,21]. Fluorescent probes have also found application in the real-time monitoring of drug delivery systems, which provide important information on drug transport and drug distribution in the body, and thereby increasing the quality of medical treatment [22,23,24]. In the last decade, the area of fluorescence sensing compounds was extended toward more complex molecular logic gates containing multiple recognition units with multiple signaling outputs [25,26,27]. Molecular logic gates are intelligent sensory molecules that analyze more than one analyte by mimicking electronic logical elements in the computer. These molecules are able to perform different sensor functions simultaneously and can calculate autonomously a composite result, which is why some scientists define them as molecular-level labs [28]. The information processing by Boolean Logic from a molecular device can be particularly useful for the rapid screening of medical conditions in which the analysis is not performed by a physician, but by the molecular device itself. Such molecular devices can save time for doctors and are crucial when health services are in extreme conditions, for example, in epidemics or bioterrorism [29,30].

**Scheme 1:**
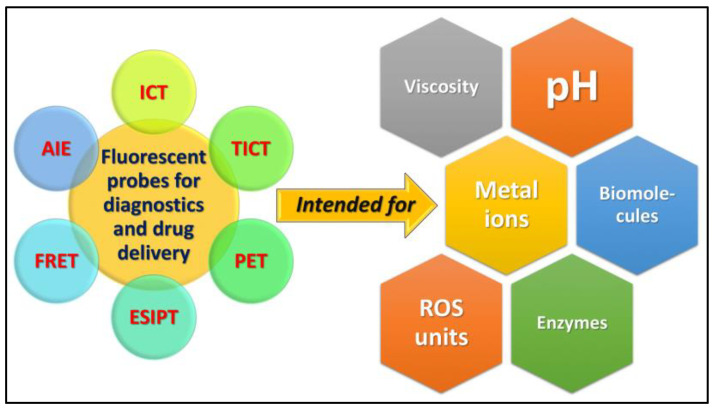
Schematic representation of the structure of the review.

The recent advances in the design and synthesis of fluorescent probes for diagnostic purposes and monitoring in drug delivery systems are discussed in several reviews [31,32,33,34,35]. Generally, they have been studied from a diagnostic point of view or described as complex nanoparticles. In this short review, we focus our attention on the main photophysical mechanisms and approaches that found application in the rational design of small fluorescent probes with high potential in the diagnostic of medical conditions and drug delivery. The photophysical phenomena Intramolecular Charge Transfer (ICT), Twisted Intramolecular Charge Transfer (TICT), Photoinduced Electron Transfer (PET), Excited-State Intramolecular Proton Transfer (ESIPT), Fluorescent Resonance Energy Transfer (FRET), and Aggregation-Induced Emission (AIE) are well described as the most popular basis for fluorescence sensing and imaging (Scheme 1).

The basic strategies in fluorescent probes for in vivo and in vitro visualization are illustrated with examples for the detection of pH, biologically important cations and anions, reactive oxygen species (ROS), viscosity, biomolecules, and enzymes, which currently are very attractive for diagnostic purposes (Alzheimer’s disease, cancer, and diabetes). In addition, an overview of recent achievements in the development of fluorescence probes as molecular logic devices with biomedical application was provided. Moreover, the implementation of the above photophysical phenomena in fluorescence–drug conjugates for theranostic and drug delivery systems is revealed. The structure of the present review can be summarized according to Scheme 1.

## 2. Fluorescent Probes Based on Intramolecular Charge Transfer (ICT)

**Figure 1:**
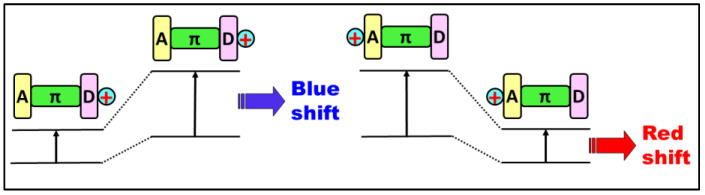
ICT probe based on cation interaction with an electron-donating or electron-withdrawing group.

The phenomenon, intramolecular charge transfer (ICT), occurs after light absorption of fluorophoric systems in which an electron-donating unit was directly conjugated with an electron-withdrawing one [36,37]. During excitation, such systems undergo donor–acceptor charge transfer from the donor to acceptor that results in “charge-polarized” excited states with a positively charged electron-donor site. The subsequent change in the dipole moment leads to a Stokes shift, which is strongly microenvironmentally dependable. This makes ICT systems a very suitable platform for sensing designing, as the presence of a charged guest nearby the donor or acceptor moiety changes the ICT efficiency and, respectively, the fluorophore photophysical properties. For example, when a cation interacts with the electron-donor group in an ICT system, it reduces the electron-donating ability of the donor. Due to the resulting reduction in conjugation, a blue shifting of the absorption is predicted together with a reduction in the extinction coefficient (Figure 1, left side). Interaction of the cation with the ICT acceptor group results in the opposite effect owing to the enhanced electron-withdrawing character of the acceptor moiety; thus, the absorption spectrum is red-shifted, and the molar absorption coefficient increases (Figure 1, right side). In principle, the fluorescence spectra are shifted in the same direction as the corresponding absorption spectra. Additionally, changes in the quantum yields and lifetimes are also expected, as the observed effects strongly depend on the charge and the size of the cation.

**Figure 2:**
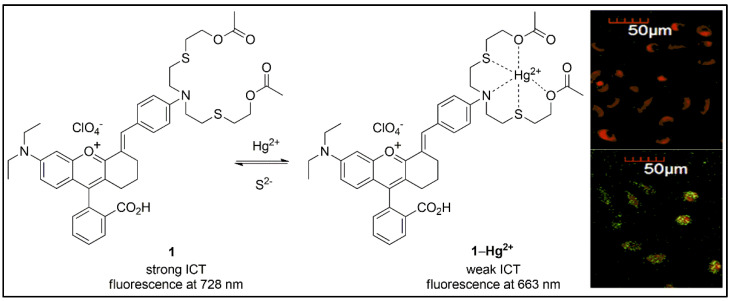
Fluorescence recognition mechanism and reversibility of probe **1** with Hg^2+^ ion. Fluorescence image of L929 cells incubated with probe (5 μM) after staining for 30 min (upper panel) and after incubated with Hg^2+^ (50 μM) for another 30 min (lower panel). Adapted with permission from [38]. Copyright (2019) Elsevier.

A typical ICT-based probe for metal cations is compound **1**, which possesses a chelate unit NS_2_O_2_ for the highly sensitive and selective detection of Hg^2+^ ions (Figure 2). This system showed a fluorescent signal at 728 nm due to the strong ICT occurring from the electron-donating nitrogen in the chelate moiety to the chromenylium-cyanine fluorophore [38]. In presence of Hg^2+^, the fluorescence of **1** was blue-shifted to 663 nm due to the lowered ICT efficiency as a result of the Hg^2+^ chelating process in which the lone electron pair on the nitrogen participated. This observation has several advantages for the monitoring of living organisms and it was successfully applied for real-time detection of Hg^2+^ in living cells. First, probe **1** emitted in a near-infrared (NIR) region where the biomolecules have weak light absorption and fluorescence that reduce background interference such as auto-fluorescence, scattering, and absorption by biomolecules. In addition, the NIR has a good penetrating ability, allowing deep penetration in living systems. Moreover, the resulting fluorescence changes at 728 nm and 663 nm in **1** allow a ratiometric signaling output. In ratiometric methods, the presence of an analyte is quantified by the ratio of fluorescent intensities of two different fluorescent bands, which provides a self-calibration effect and built-in correction for environmental effects including biomolecules. Additionally, probe **1** showed good cell permeability and low toxicity.

**Figure 3:**
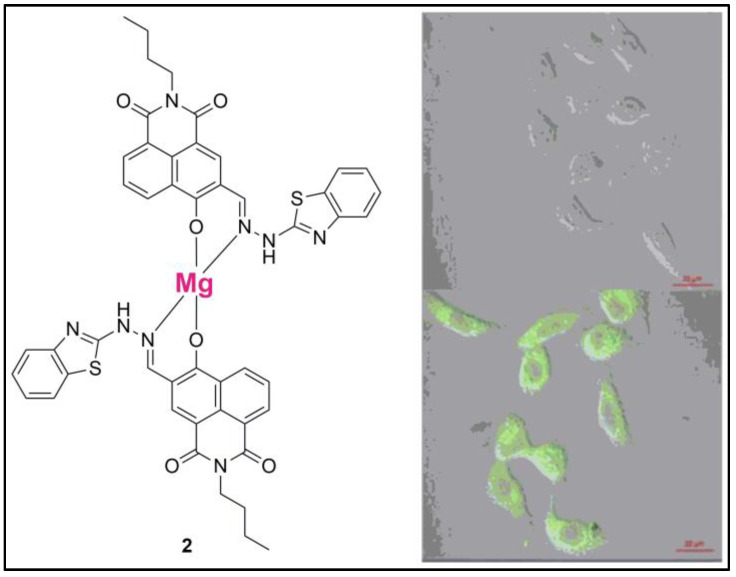
Proposed sensing process of probe **2** for Mg^2+^. Fluorescence image of A549 cells incubated with probe (10 μM) for 30 min (upper panel) and then incubated with 2 μM Mg2+ for 30 min (lower panel). Adapted with permission from [37]. Copyright (2017) Elsevier.

Intracellular Mg^2+^ plays important role in physiological processes such as activities of ion channels in membrane proteins and the regulation of many enzymes. The abnormal concentration of intracellular Mg^2+^ is associated with renal losses, neurological manifestations, diabetes, osteoporosis, and coronary heart disease [39,40,41]. That is why the detection of intracellular Mg^2+^ is very attractive from a diagnostic point of view. The 1,8-naphthalimide-based probe **2** is an interesting example of a fluorescent probe for the selective intracellular imaging of Mg^2+^ [42] (Figure 3).

It is well-known that the light absorption of 1,8-naphthalimide fluorophore results in a charge transfer interaction between the C-4 positioned substituent and both the electron-accepting carbonyl groups, whose efficiency strongly depends on the electron-donating ability of the C-4 substituent [43,44,45]. Thus, the decreased electron-donating capability of the hydroxyl group bounded at the C-4 position of **2** after the binding process with Mg^2+^ reduced the ICT in this probe and resulted in a blue shift in its absorption from 480 to 393 nm. It was found that the changes in the absorption spectra were accompanied with the appearance of bright green fluorescence that was applied for selective “off-on” Mg^2+^ sensing purposes. The detection limit of 5.01 × 10^−8^ M was calculated and a 1:2 complex ratio between Mg^2+^ and the probe was determined. The formed complex was identified and characterized using ^1^H NMR, ^13^C NMR, and ESI-MS techniques. Furthermore, probe **2** showed low toxicity and good permeability in living A549 cells.

**Figure 4:**
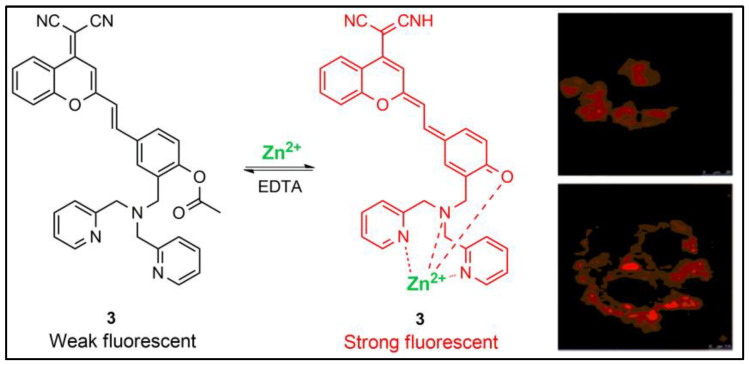
Fluorescence recognition mechanism and reversibility of probe **3** with Zn^2+^ ion. Fluorescence image of MCF-7 cells pretreated with probe (10 μM) for 30 min (upper panel) and after incubated with Zn^2+^ (100 μM) for 1 h (lower panel). Adapted with permission from [46]. Copyright (2020) Elsevier.

Zn^2+^ is another important metal cation whose intracellular detection could be useful for diagnostic purposes. This ion is involved in pathological processes, such as Alzheimer’s disease, epilepsy, ischemic stroke, infantile diarrhea, apoptosis, disorder of enzyme regulation, and neurotransmission. Recently, Ahmed et al. synthesized compound **3** as a useful ICT-based fluorescent probe for intracellular measurements of Zn^2+^ [46]. This probe showed “turn-on” NIR fluorescence emission at 661 nm in the presence of Zn^2+^, which the authors explained was due to the increased ICT efficiency in the probe after Zn^2+^ complexation (Figure 4). The resulting complex was characterized by ^1^H NMR, ^13^C NMR, and HR-MS. The probe showed low toxicity and a good signaling response upon the intracellular imaging of Zn^2+^ in MCF-7 living cells.

**Figure 5:**
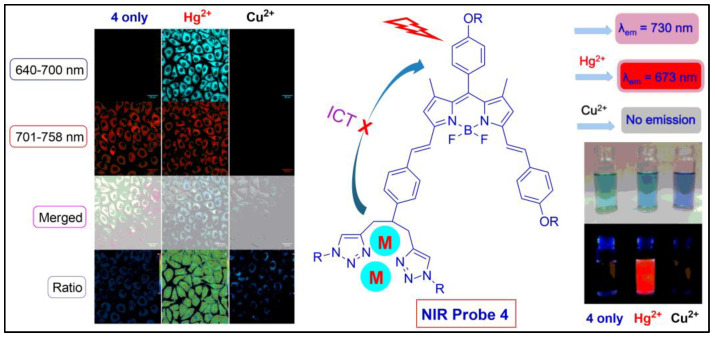
Fluorescence recognition mechanism of probe **4** with Hg^2+^ and Cu^2+^ ions. Fluorescence images of Hella cells pretreated with probe (6 μM) for 30 min and after incubated with Hg^2+^ (120 μM) or Cu^2+^ (120 μM) for 0.5 h. Adapted with permission from [47]. Copyright (2019) Elsevier.

The discriminative detection of two or more metal cations at the same time could play an essential role for better diagnostics in medicine and biology. This encouraged Huang et al. to synthesize probe **4**, which was able to coordinate Cu^2+^ and Hg^2+^ [47]. Probe **4** was designed as an ICT-based NIR fluorescence ratiometric probe, which makes it a promising candidate for real-time imaging in living organisms (Figure 5). This probe has a bright emission at 730 nm and contains an electron-donating amino group as a metal recognition unit that takes place in a strong ICT process during excitation. In the presence of Hg^2+^, the probe fluorescence was blue-shifted to 673 nm due to the binding of Hg^2+^ with electron-donating amines that reduced the ICT efficiency. Based on the observed fluorescent changes, a ratiometric analysis was constructed, which showed a value of 5.23 in the presence of Hg^2+^, and 0.37 in its absence. In addition, this probe was able to coordinate Cu^2+^ but it was found that the observed complex was non-fluorescence. Furthermore, the probe was applied in Hella cells for discriminative fluorescence detection of Hg^2+^ and Cu^2+^ ions in biological systems. The intracellular imaging of Hg^2+^ showed high emissions at the window of 640–700 nm and low emissions at NIR in the range of 701–758 nm. The presence of Cu^2+^ resulted in no emissions at both fluorescence windows. The simultaneous presence of Hg^2+^ and Cu^2+^ ions led to low emissions at 640–700 nm and no emissions at 701–758 nm.

Intracellular pH plays a critical role in physiological and pathological processes such as cell growth and apoptosis, ion transport, homeostasis, and enzymatic activity. Abnormal pH values are associated with inappropriate cell function in some common disease types such as cancer and Alzheimer’s. Hence, the monitoring of intracellular pH has attracted increasing interest.

**Figure 6:**
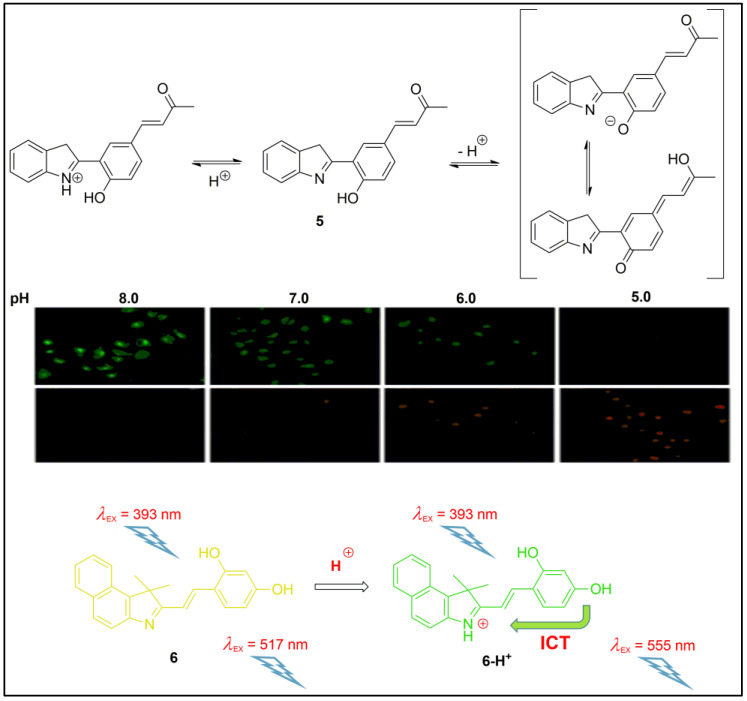
Acid–base equilibrium of probes **5** and **6**. Fluorescence images of MCF-7 cells pretreated with probe **5** (5 μM) for 2 h and after incubated using PBS with various pH values for 20 min. The images are collected at 470–545 nm (upper row) and 545–650 nm (lower row). Adapted with permission from [48]. Copyright (2017) Elsevier.

The illustrated Figure 6 fluorescence compounds **5** and **6** were rationally designed as probes for measurements of intracellular pHs using a very similar strategy [48,49]. Both systems are ICT fluorophores in which the electron-accepting part is a nitrogen-containing heterocycle—in **5**, it is benzothiazole and in **6**, it is benzo[e]indole. In neutral form, these molecules possess green fluorescence owing to the occurrence of ICT in an excited state. In acid media, the probes change their green emissions in red fluorescence due to the protonation of the electron-accepting heterocyclic nitrogen that enhanced its electron deficiency and ICT efficiency, respectively. The maximal fluorescence intensity in **5** was shifted from 515 to 565 nm. This change was about 10 nm more than the one in **6**, where the fluorescence maximum was shifted from 517 to 555 nm. Based on the observed changes, a ratiometric analysis was obtained and implemented for pH measurements in living cells. However, due to the different acidity of both receptors, the **5** and **6** were suitable for pH determination at different pH windows. Compound **5** showed a p*K*_a_ value of 6.73 and fluorescence response from pH 7.5 to pH 6.0, while the p*K*_a_ of probe **6** was calculated to be 4.98, which makes it useful for pH measurements ranging between 5.5 and 4.6. Due to the higher acidity, **6** was applied for the detection of pHs in lysosomes, which are known as acid organelles.

**Figure 7:**
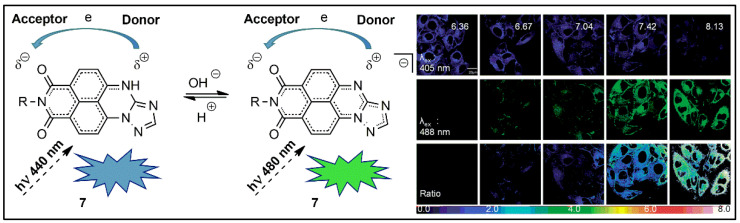
Sensing process of probe **7**. Confocal fluorescence images of MCF–7 cells incubated in high K+ HEPES-buffered solution containing ionophores at different pHs. Reproduced with permission from [50]. Copyright (2012) The Royal Society of Chemistry.

The condensation of 3-amino-1,2,4-triazole to the C-4 and C-5 position of the 1,8-naphthalimide fluorophoric system resulted in an interesting architecture suitable for the ratiometric intracellular imaging of pH [50,51,52]. The introduced aminotriazole in 1,8-naphthalimide fluorophore has unusually high acidity compared to other 1,8-naphthalimide probes and showed a p*K*_a_ value in the physiological range of about 6.5. Additionally, the aminotriazole naphthalimides showed increased water solubility, low cytotoxicity, clear cellular location, and membrane permeability making them an excellent platform for pH measurements in living cells [50]. In a slightly acid and neutral solution, the condensed aminotriazolyl-1,8-naphthalimide fluorophore **7** was blue-emitting and showed a fluorescence maximum at 440 nm due to the ICT interaction of aromatic amine at C-4 position to the electron-accepting imide (Figure 7).

At slightly alkaline media, this amine **7** was deprotonated and formed an anion that strongly increased the electron density of the substituent at the C-4 position. As a result, the ICT efficiency in the probe was enhanced and the former fluorescence at 440 nm was red-shifted to 480 nm. Zhou et al. have illustrated that the long chain-containing derivatives can be located on the membrane structure of the cells and could be used for the pH mapping of both the extracellular microenvironment and inner cells by confocal imaging [50].

Moreover, based on the ratiometric pH response to the tumor microenvironment, the condensed aminotriazolyl-1,8-naphthalimide **7** was successfully used to image the pH of subcutaneous tumor xenografts [51].

**Figure 8:**
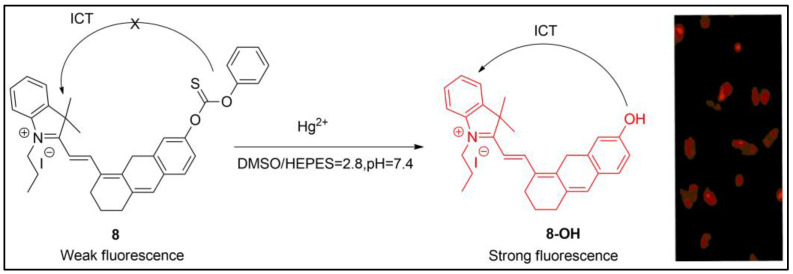
Response mechanism of probe **8** to Hg^2+^. A549 cells incubated with **8** (10 μM) for 30 min, and continually incubated with Hg^2+^ (100 μM) for 60 min. Adapted with permission from [53]. Copyright (2020) Elsevier.

The reversible coordination is not the only approach for interaction between the probes and analytes. An alternative way is the reactive-based probes in which a selective chemical reaction between the recognition part in the receptor and the analyte occurs. The general advantage of the reactive probes is their selectivity that was induced from a specific reaction with the analyte. A typical example of a reactive ICT probe is presented in Figure 8. Probe **8** represents a conjugated system containing two electron-accepting fragments—indole and thiocarbonyl units [53]. The thiocarbonyl was introduced as a selective Hg^2+^ receptor moiety due to the thiophilic characteristics of that cation. After desulfurization in the presence of Hg^2+^, electron-accepting thiocarbonyl in **8** was replaced with a strong electron-donating hydroxyl group; thus, an ICT interaction was raised, and a NIR fluorescent emission at 708 nm was observed. Moreover, it was found that the probe was biocompatible, organism-permeable, and was used for the detection of intracellular Hg^2+^ Cy-PT cells and zebrafish.

**Figure 9:**
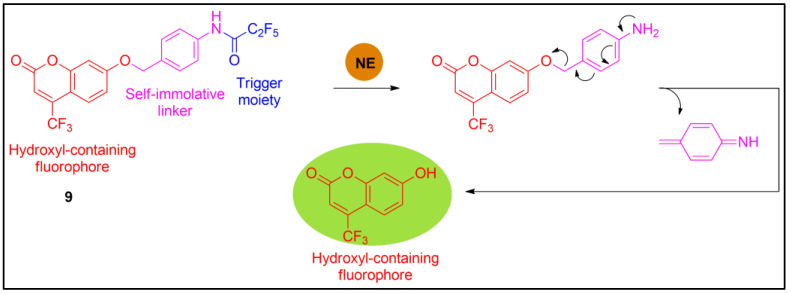
Construction of NE probe **9** containing hydroxyl fluorophores and self-immolation group. Adapted with permission from [54]. Copyright (2023) Elsevier.

Li et al. used the same strategy for the real-time and specific detection of neutrophil elastase in living cells [54]. Overexpression of neutrophil elastase may degrade some proteins leading to serious lung diseases such as lung injury, chronic obstructive lung disease, acute respiratory, and distress syndrome. In addition, neutrophil elastase can promote tumor growth and metastasis, which indicates lung cancer in tumor tissue [55,56,57,58]. Therefore, the detection of neutrophil elastase in living organisms could be used as a critical biomarker for the diagnosis of lung diseases and lung cancer. Probe **9** was designed as a new strategy for the construction of a neutrophil elastase fluorescent probe [54]. It contained pentafluoropropionyl as a receptor fragment that was bounded to a 4-trifluoromethyl-7-hydroxyl coumarin fluorophore (Figure 9).

Due to the lack of donor–acceptor interaction, the ICT process in the probe was not feasible and its fluorescence emission was not observed. However, in the presence of neutrophil elastase, the receptor fragment was hydrolyzed to a hydroxyl group and probe **9** was converted to 4-trifluoromethyl-7-hydroxyl coumarin with a strong ICT nature giving green fluorescence. Due to the use of the pentafluoropropionyl receptor part, the novel probe **9** showed high selectivity, ultra-high fluorescence enhancement, and a high signal-to-noise ratio compared to the previously reported probe, containing esters and amides as recognition units. Furthermore, **9** showed good biocompatibility and low toxicity. Thus, the probe was successfully applied for the selective real-time detection of neutrophil elastase (NE) activity in living HeLa, SKOV3, A549, and HepG2 cells, and zebrafish.

The lack of oxygen in tissues is known as hypoxia. With hypoxia stress, excessive reactive oxygen species (ROS) are produced, which results in cell damage and death. In terms of biological reagents, types of ROS are superoxide anion (O_2_^−^), hydrogen peroxide(H_2_O_2_), lipid peroxides (ROOH), hypochlorous acid (HClO), singlet oxygen (^1^O_2_), ozone (O_3_), and peroxynitrite (ONOO-), which are constantly used in redox activities of living systems. Recent research has revealed that cancer cells have approximately ten times higher concentrations of ROS compared to normal cells; thus, ROS-responsive probes could be a promising platform for the discriminative detection of cancer cells over normal cells.

**Figure 10:**
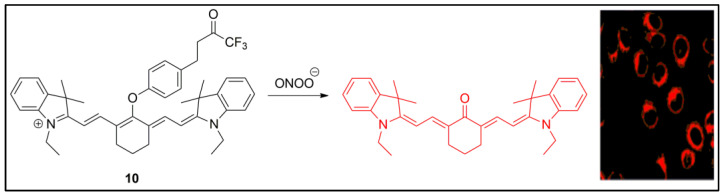
Probe **10** detection mechanism proposed for ONOO. Imaging of LO2 cells treated with probe **10** (10 μM) for 30 min under hypoxic conditions with 1% O_2_. Adapted with permission from [63]. Copyright (2022) Elsevier.

ONOO- is a ROS unit that is produced in living organisms by O_2_^−^ and nitric oxide (NO). Its excessive cell generation is associated not only with cancer but also with many other disorders such as inflammatory diseases, diabetes, sepsis, atherosclerosis, Alzheimer’s, and other neurodegenerative diseases [59,60,61,62]. Huang et al. synthesized probe **10**, designed for the selective and discriminative detection of hypoxia cells over normal cells [63]. Probe **10** consisted of the 1,1,1-Trifluoro-4-(4-oxyphenyl)butan-2-one group as the recognition group and a heptamethine cyanine dye as the NIR fluorescence reporter. In the presence of ONOO-, this probe was oxidized and released a strong ICT heptamethine cyanine fluorophore with maximal fluorescence at 630 nm (Figure 10). The observed detection limit was 9.2 nM. The probe showed low toxicity, great biocompatibility, and was successfully applied to detect hypoxia injury in Cy-CF3, zebrafish, and hypoxic mice.

**Figure 11:**
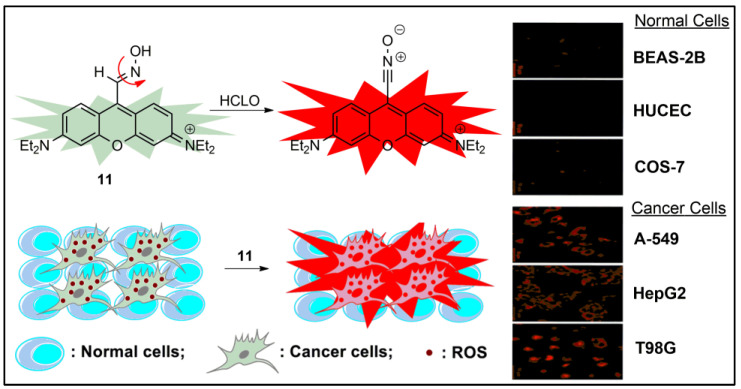
Proposed reaction mechanism of probe **11** for HClO and Schematic illustration of discriminating cancer cells from normal cells using **11**. Fluorescence images of **11**-loaded (2 μM, 20 min) cancer cells and normal cells. Adapted with permission from [64]. Copyright (2021) Elsevier.

Wang et al. rationally designed probe **11** as an HClO fluorescence sensing compound for the discriminative detection of cancer cells based on selective ROS reactive interaction (Figure 11) [64]. In a simulated physiological condition, the probe showed a rapid NIR fluorescence off–on response (response time: 10 s) toward HClO as an endogenous ROS species with a detection limit of 2.4 nM. The HClO triggered an efficient oxidation chemical process of the aldoxime recognition group to nitrile, resulting in an ICT-based fluorophoric system with maximal emissions placed at 680 nm. Probe **11** showed negligible cytotoxicity, high sensitivity, and has been applied to discriminate cancerous cells and tissues from normal ones.

Superoxide anion (O_2_^−^) is a ROS unit that produces considerably less than 1-2% of consumed oxygen molecules in the mitochondrial respiratory chain, and the diffusing superoxide anion typically is converted to other ROS (such as hydrogen peroxide, peroxynitrite, and perhydroxyl radical) in biological systems. An excessive level of superoxide anion induced increasing oxidative stress and mitochondrial dysfunction that could be an indication of diabetes rheumatoid arthritis, heart disease, Alzheimer’s disease, and cancer [65,66,67,68,69,70].

**Figure 12:**
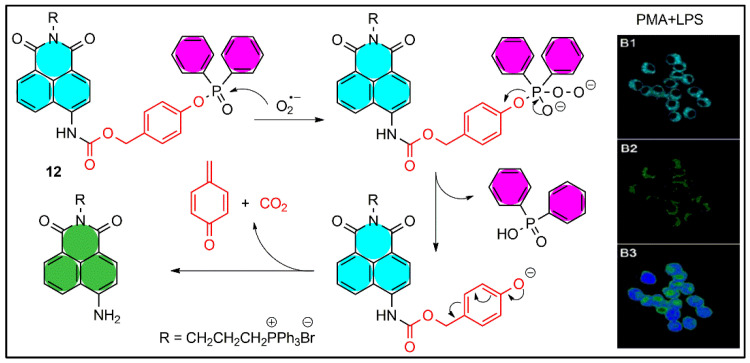
Proposed sensing mechanism of **12** toward O_2_^•−^. Fluorescence imaging with endogenous O_2_^•−^ using probe **12** (2 μM) for 30 min. RAW264.7 cells were treated with PMA/LPS and stained with **12**. Adapted with permission from [71]. Copyright (2018) American Chemical Society.

This focused attention of Zhang et al. to prepare mitochondria-accessing ratiometric fluorescent probe **12** capable of detecting selectively superoxide anions [71]. As a receptor fragment in probe **12**, a triphenylphosphonium unit was introduced in which the diphenylphosphinate part trigged selective oxidation in the presence of superoxide anions. Because of the oxidation of **12**, a green emitting 4-amino-1,8-naphthalimide was obtained (Figure 12). The green fluorescence was attributed to the enhanced ICT in the 1,8-naphthalimide fluorophore containing strong electron-donating amines instead of a C-4 carbamate group in the former blue-emitting naphthalimide **12**. Using the fluorescent changes at 475 nm and 540 nm, a ratiometric analysis was constructed and applied for the detection of mitochondrial superoxide anions as well as inflammatory D magna. The limit of detection was determined to be 0.370 μM. The cytotoxicity test revealed more than 80% cell viability, suggesting that the probe can be used safely in living cell imaging.

**Figure 13:**
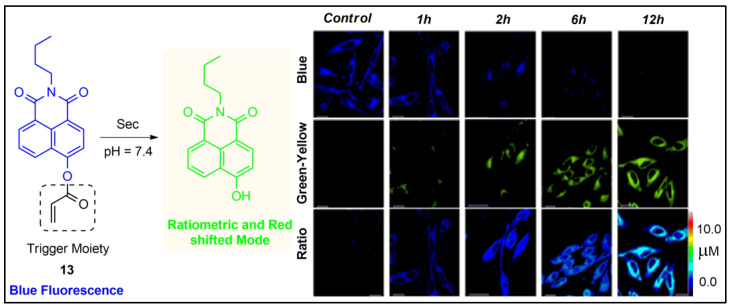
Proposed sensing mechanism for Sec activation of **13**. The in situ fluorescence changes of Hela cells stimulated to Na_2_SeO_3_ (5 μM) for different times (1, 2, 6, 12 h), and then incubated with **13** (5 μM). Adapted with permission from [72]. Copyright (2017) The Royal Society of Chemistry.

Monitoring intracellular selenocysteine (Sec) is of significant interest for studying Sec metabolism and its changes in disease-relevant concentrations. The 1,8-naphthalimide **13** was prepared as a selective probe for Sec imaging by ratiometric fluorescence response [72]. The 1,8-naphthalimide probe **13** was a blue-emitting ICT system with acrylate moiety at position C-4 (Figure 13). At physiological conditions, in the presence of selenocysteine, the probe undergoes hydrolysis to 4-hydroxy-1,8-naphthalimide and changes its initial blue emission from blue (fluorescence maximum at 420 nm) to green (fluorescence maximum at 546 nm) due to the increased electron ability of C-4 substituent and enhanced ICT process. Based on the observed changes, a ratiometric analysis with a detection limit of 12 nM selenocysteine was observed. Furthermore, probe **13** was applied for the high specificity and selectivity rapid quantification of exogenous and endogenous Sec in living Hela cells.

**Figure 14:**
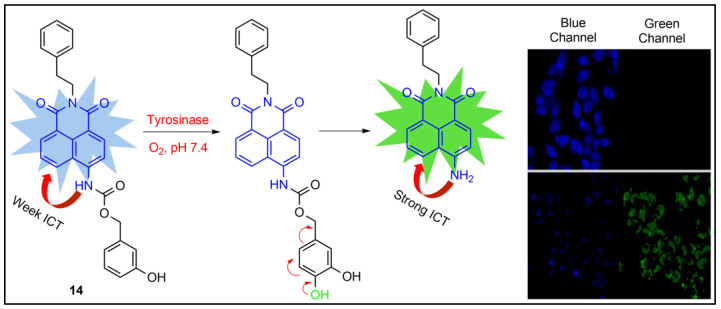
Tyrosinase detection strategy. Fluorescence images of A375 after 30 min of incubation with probe **14** (upper panel). Cells exhibit strong blue emissions compared to green channel suggesting that the probe was not significantly catalyzed by endogenous tyrosinase. After 3 h of incubation (lower panel), green emissions of A375 enhanced prominently while blue emissions quenched significantly. Green emissions indicate the endogenous catalysis of probe **14**. Adapted with permission from [76]. Copyright (2018) The Royal Society of Chemistry.

Tyrosinase is a polyphenolic oxidase enzyme with clinical importance due to its association with the progress of some diseases such as Parkinson, dopamine neurotoxicity, schizophrenia, and melanoma cancer [73,74,75]. This encouraged Sidhu et al. to obtain the 1,8-naphthalimide **14** as an efficient fluorescent ratiometric probe for intracellular imaging of tyrosinase (Figure 14) [76].

The probe was designed as an ICT naphthalimide fluorophore, conjugated with a 3-hydroxybenzyl unit at the C-4 position as a receptor for selective tyrosinase reaction. Because of the low ICT process in **14**, the probe showed blue emissive fluorescence output at 467 nm. In the presence of tyrosinase at physiological conditions **14** was hydrolyzed to 4-amino-1,8-naphthalimide containing a strong electron-donating group, which increased the ICT efficiency and red-shifted the former probes’ emissions to the green region at 535 nm. It was found that the probe displayed a sensitive ratiometric response to tyrosinase with a detection limit of 0.2 U mL^−1^. The MTT analysis showed moderate cytotoxicity—70% cell viability at 20 μM. In addition, **14** was used to image endogenous tyrosinase in A375 cells in the blue and green channels of a confocal microscope.

**Figure 15:**
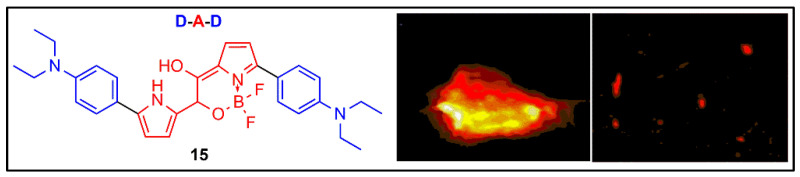
Fluorescence image of the AD model mice (left panel) and in vitro fluorescence staining of **15** on age-matched Tg mouse (right panel)—APP/PS1 transgenic, male. Adapted with permission from [77]. Copyright (2022) Elsevier.

The ICT donor–acceptor–donor mode is an interesting approach for the design of an NIR probe for bioimaging. An example is illustrated in Figure 15, where two electron-donating *N*,*N*-diethylaniline recognition groups were bonded to a single boron difluoride bridged azafluvene as the strong electron-withdrawing group in order to obtain NIR fluorescent probe **15** for Amyloid β-peptide [77]. Amyloid β-peptide (Aβ) aggregation is a primary biomarker for Alzheimer’s disease; hence, the preparation of a fluorescent probe for its detection plays an important diagnostic role. It was found that the *N*,*N*-diethylaniline recognition groups were selectively binding Aβ oligomers and were not responsive to other biomolecular small molecules. The resulting complex was strongly sensitive to the Aβ aggregation process due to the environmentally sensing ICT nature of **15** and showed off–on emissions during the agglomeration of Aβ. Probe **15** showed low cytotoxicity toward HaCat, HeLa, and bEnd.3 cells, and it was used for a brain-imaging Alzheimer’s disease model mice. The observed results illustrated the great potential of **15** for early in vivo diagnosis of Alzheimer’s disease.

## 3. Fluorescent Probes Based on Twisted Intramolecular Charge Transfer (TICT)

**Figure 16:**
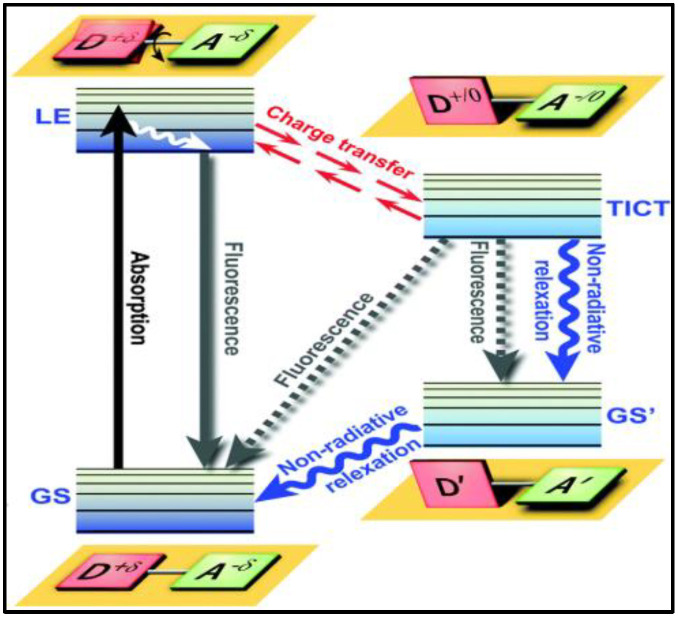
Twisted intramolecular charge transfer (TICT) dynamics. Upon excitation from the GS, the LE state equilibrates rapidly with the TICT state after fast charge transfer (GS = ground state; LE = locally excited state). Adapted with permission from [78]. Copyright (2016) The Royal Society of Chemistry.

In some cases, the excitation of ICT systems induces an intramolecular rotation in which the donor and acceptor parts take more stable coplanar conformation in an excited state. After excitation to the Twisted Intramolecular Charge Transfer (TICT) state, the chromophoric system returns to a ground-state radiative level through red-shifted emissions or by non-radiative relaxation (Figure 16) [78].

The observed fluorescence properties are environmentally dependent; thus, the TICT fluorophores are the ideal platform for the construction of fluorescent probes. Notably, the TICT excited state is strongly solvent-viscosity dependable due to the prevented bond rotation in more viscous liquids and makes this phenomenon especially valuable for imaging microenvironmental viscosity in biological objects [79,80,81].

**Figure 17:**
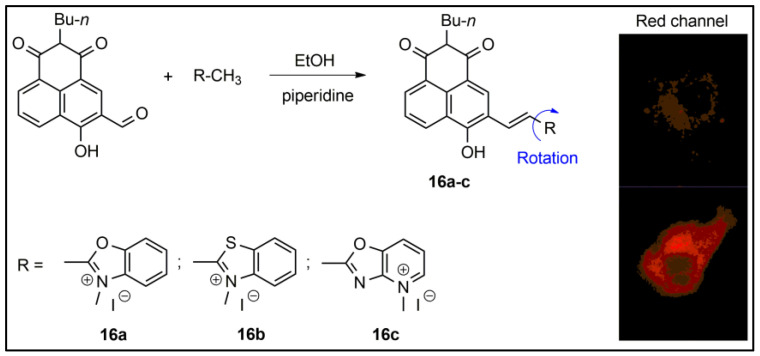
Synthetic routes of probes **16a-c**. Confocal laser fluorescence images of probe **16a** in HeLa cells. Cells incubated with the probe (10 μM) for 20 min (upper panel) and incubated with monensin (10 μM) for 30 min and probe **16a** (10 μM) for another 20 min (lower panel). Adapted with permission from [82]. Copyright (2021) Elsevier.

For example, recently Wei et al. prepared three 1,8-naphthalimides **16a-c** bounded to different nitrogen-containing heterocycles (Figure 17) [82]. These compounds showed only very weak fluorescence emissions due to non-radiative decay through a twisted intramolecular charge transfer. The authors found that the probes **16a-c** showed well-pronounced fluorescence emissions at 580–630 nm in a viscose solution such as glycerol due to the hindered TICT process. The observed fluorescence enhancement from low viscose methanol to glycerol solution was by about 19 times. Moreover, all of the three probes showed low cytotoxicity against Hella cells with a calculated viability higher than 80% at 15 μM concentration, and they were used for imaging the fluctuations of the mitochondrial viscosity in Hella cells. This makes the synthesized probes a potential tool for the early diagnosis of mitochondrial-related diseases.

**Figure 18:**
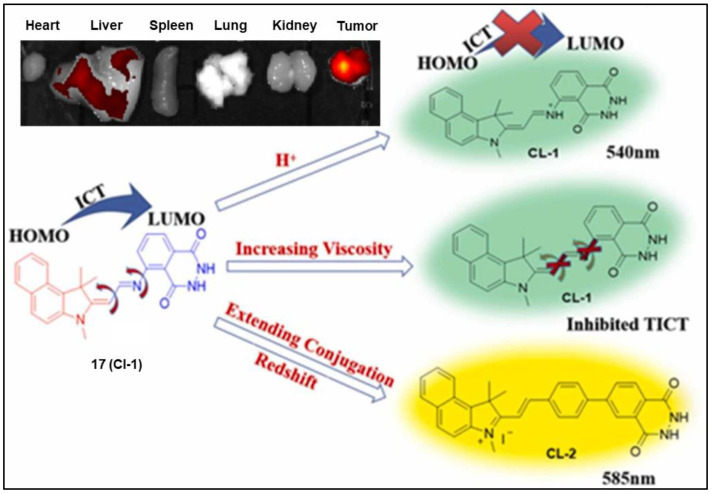
Design strategy of probe **17**. Fluorescence images of organs and tumors of tumor-bearing mice after tail vein administration of **17** (100 µM). Adapted with permission from [84]. Copyright (2023) Elsevier.

Intracellular viscosity is an index affecting physiological processes that affect the transport, diffusion, and interactions between biomolecules. The abnormal viscosity values are related to atherosclerosis, hypertension, diabetes, Alzheimer’s disease, and cancer [83]. Wang et al. applied this for highly selective tumor visualization using TICT fluorescent probe **17** (Figure 18) [84]. Similar to probes **16a-c**, compound **17** showed only weak fluorescence at 555 nm due to TICT non-emissive deexcitation. However, in glycerol, this emission was highly enhanced and the observed fluorescence enhancement calculated from the fluorescent intensities in water and glycerol was more than 200 times. Because the viscosity in cancer cells is higher than normal, the authors selectively visualized tumors in mice models. As can be seen in Figure 18, isolated, and incubated in 20 μM of the probe, solutions of sample heart, liver, spleen, lung, kidney, and tumor are presented, where the tumor shows significantly higher fluorescence over the organs. Together with these results, probe **17** showed low toxicity and good biocompatibility, which makes it an excellent tool for tumor visualization.

**Figure 19:**
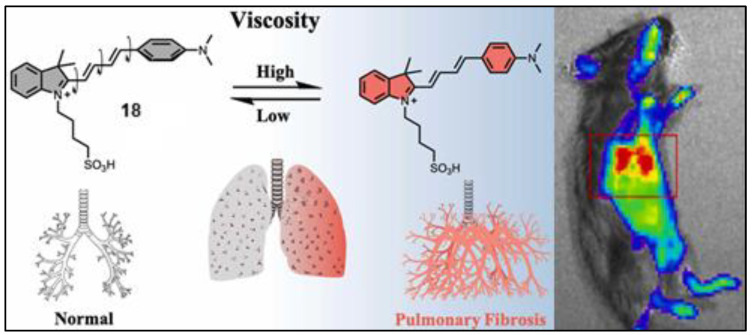
Transformation of probe **18** in monitoring viscosity fluctuation in idiopathic pulmonary fibrosis. In vivo imaging of bleomycin-induced IPF mice model in 14 days, *λ*_EX_ = 640 nm, *λ*_F_ = 700 nm. Adapted with permission from [85]. Copyright (2022) Elsevier.

Based on the fluorescence properties of NIR TICT probe **18**, Zhan et al. developed, for the first time, a fluorescent method for the early exploration of idiopathic pulmonary fibrosis through changes in viscosity (Figure 19) [85].

Probe **18** is a TICT probe with low NIR fluorescence at 703 nm, which was enhanced in a higher viscosity solution and showed fluorescence enhancement 54.4 after transitioning from water to glycerol solution buffered with PBS buffer. The probe has good water solubility, NIR fluorescence emission, low toxicity, and great biocompatibility; thus, it was applied for the monitoring of microviscosity fluctuations in living cells. More importantly, this probe confirmed that pulmonary fibrosis has a positive correlation with an increase in viscosity, and it was used for sensitive visualization in the early stage of idiopathic pulmonary fibrosis in vivo in clinical patient tissue.

**Figure 20:**
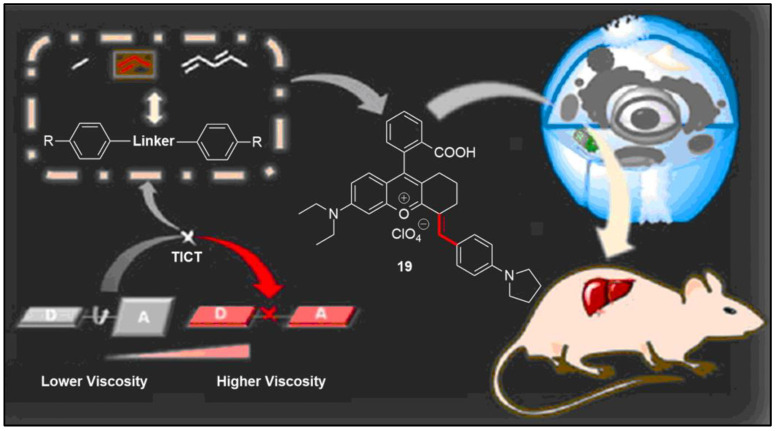
Molecular structure of probe **19** and the viscosity sensing mechanism. Adapted with permission from [86]. Copyright (2022) Elsevier.

Abnormal intracellular viscosity also causes fatty liver and hyperlipidemia. This fact was used by Song et al. to prepare a viscosity-sensing probe **19** for the detection of fatty liver at the organ level (Figure 20) [86]. The TICT nature of this molecule resulted in a low fluorescence centered at 740 nm, which was enhanced by more than 200 times in PBS-buffered glycerol compared to the PBS buffer solution. The probe showed very low toxicity at 30 μM using CCK-8. Moreover, the prevented rotation in this molecule was used to study mitochondrial viscosity mutations in living mouse fatty liver disease models, and it was found that it could distinguish fatty liver mice from normal liver mice.

Fluorescent thermometers (thermosensitive fluorescent probes) with near-infrared (NIR) emission play an important role in the real-time visualization of intracellular temperature with high resolution and the investigation of cellular functions and biochemical activities. An approach for the construction of thermosensitive fluorescence probes was based on the viscosity-sensing TICT probes due to the high dependence of viscosity toward temperature.

**Figure 21:**
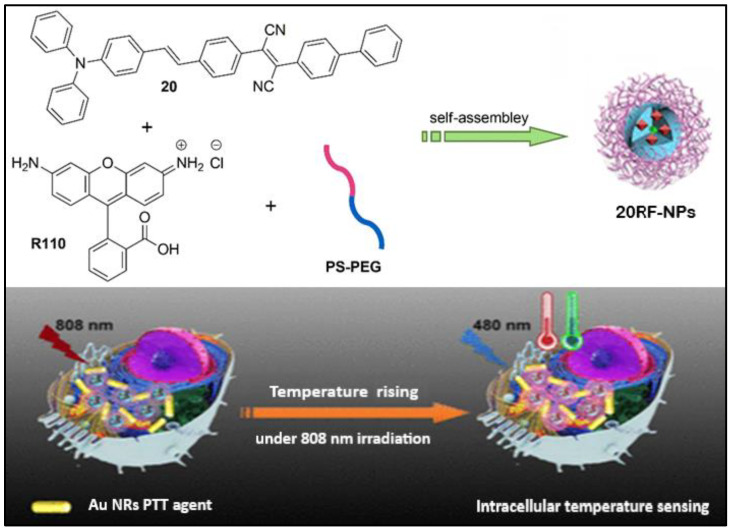
Self-assembly of probe **20** with Rhodamine 110 into amphiphilic polymer PS-PEG to form nanoparticles 20RF NPs and illustration of intracellular temperature sensing. Adapted with permission from [87]. Copyright (2020) American Chemical Society.

It is well known that the temperature increase resulted in a high flow ability and low viscosity, respectively. Based on this knowledge, Meng et al. prepared NIR fluorescent probe **20** (Figure 21) in which an effective TICT process quenched the fluorescence emission at 680 nm after decreased viscosity as a result of increased temperature [87]. Probe **20** showed low cytotoxicity to Hep-G2 cells through an MTS assay and good biocompatibility. In addition, by encapsulating thermosensitive probe **20** and Rhodamine 110 into an amphiphilic polymer matrix F127, it was applied as excellent and reversible temperature TICT fluorescent sensitive nanoparticles (20RF NPs) that could monitor cellular temperature change from 25 to 53 °C of Hep-G2 cells under photothermal therapy agent heating process. Thus, it displayed a promising potential for applications in biological temperature measurements.

## 4. Fluorescent Probes Based on Photoinduced Electron Transfer (PET)

**Figure 22:**
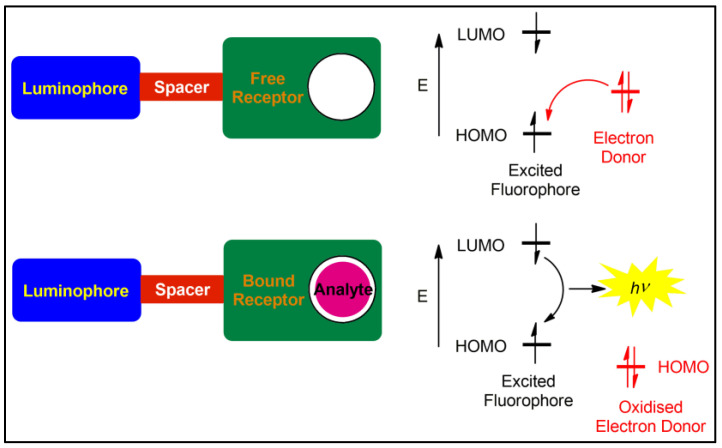
PET mechanism.

The photoinduced electron transfer (PET) process became one of the most popular approaches for the construction of chemosensing probes, since the works by A.P. de Silva who developed the “*fluorophore*–*spacer*–*receptor*” model as rational design of fluorescent sensing systems [88,89,90,91,92]. This model was distinguished by simple construction and easier and more predictable communication between the receptor (recognition part) and the fluorophore (signaling part). The system consists of a fluorophore linked to an electron-rich receptor fragment (usually amino nitrogen). The fluorophore is a photonic transactions site of excitation and emission, which produces the fluorescence signaling output. The receptor is responsible for guest complexation and decomplexation. A spacer holds the fluorophore and receptor close to each other, but in separated conjugated systems. After excitation of the fluorophore, an electron transfer occurs from the HOMO of the electron-donating receptor to the low-lying HOMO of the electron-accepting fluorophore. Thus, fluorescence does not take place. When a cation binds to the recognition moiety, the energy of the HOMO of the receptor is lowered so that the photoinduced electron transfer cannot happen from the HOMO of the donor to the fluorophore (Figure 22). This results in a bright fluorescence emission. In other words, the presence of a guest is signalized with the enhanced quantum yield of fluorescence, and respectively, fluorescence intensity.

**Figure 23:**
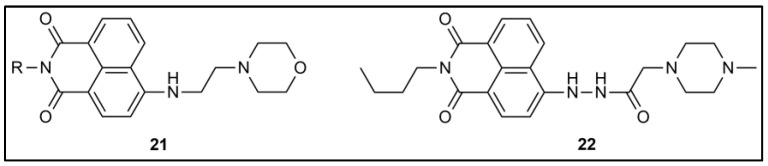
Naphthalimides **21** and **22** containing morpholine and *N*-methylpiperazine, respectively [95,96].

The incorporation of trialkyl amines as PET receptor fragments in common fluorophores such as 1,8-naphthalimide and perylenediimide is a classic platform for the design of pH-sensitive fluorescent probes. However, the tertiary alkyamino containing PET probes showed a p*K*_a_ value of about nine, which makes them inappropriate for the determination of pH fluctuations in a physiological range [93,94]. An approach to shifting these values in the physiological range was the use of morpholine or *N*-methylpiperazine as receptors, which, for instance, in PET probes **21** and **22**, resulted in pKa = 6.4 and pKa = 6.3, respectively (Figure 23) [95,96].

**Figure 24:**
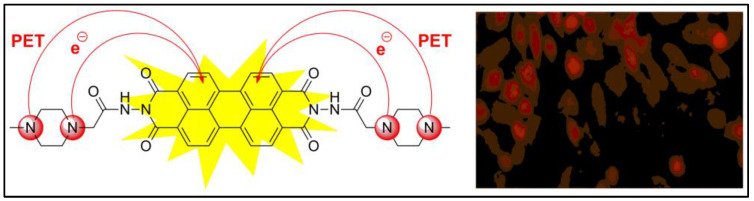
Perylene-3,4,9,10-tetracarboxylic diimide-based probe **23**. Adapted with permission from [97]. Copyright (2019) Elsevier.

Additionally, morpholine or *N*-methylpiperazine increased water solubility, which is a serious benefit for the needs of modern biomedical applications. This encourages us to synthesize perylenediimide **23** as an efficient PET-based probe (Figure 24) [97]. Probe **23** showed unusually high water solubility as a perylenediimide derivative and selective PET off–on fluorescent response toward pH. In aqueous solution, the prevented PET quenching process in acid media resulted in bright fluorescence at 550 nm as the fluorescent quantum yield enhanced from 0.003 to 0.11. In addition, the observed p*K*_a_ value of 6.35 in **23** made it a suitable probe for pH determination in the physiological range. Probe **23** showed good cell permeability in L929 cell lines and according to the MTT analysis; it was low cytotoxic.

**Figure 25:**
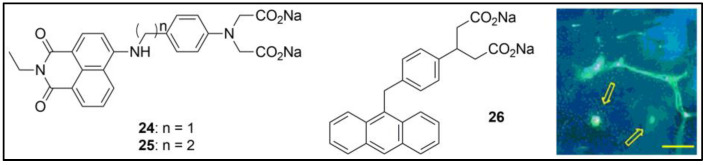
Structures of PET sensors **24**–**26**. Transverse section of the bone labeled with **24**, viewed in UV epifluorescence (365 nm); yellow arrow shows osteons with its lacunae, Haversian canal, and canaliculi. Bar = 100 μm. Adapted with permission from [98]. Copyright (2009) Elsevier.

Gunnlaugsson et al. used the “*fluorophore*–*spacer*–*receptor*” format in compounds **24**–**26** to obtain fluorescent probes for the visualization of microdamage in bones such as cracks and scratches [98]. Probes **24**–**26** possessed phenyliminodiacetate as a receptor unit that can bind Ca^2+^ ions selectively and completely quenched the fluorophore emissions. These effects were applied for the visualization of cracks and scratches on bones that generated Ca^2+^ sites on the bone surface capable of binding with the exposed probes **24**–**26**, leading to a bright fluorescent emission because of the hindered PET. It was found that the blue-emitting probe **26** was masked by the autofluorescence from the bone matrix, while the binding of both **24** and **25** gave significant fluorescence arising from the 4-amino-1,8-naphthalimide fluorophore (Figure 25).

**Figure 26:**
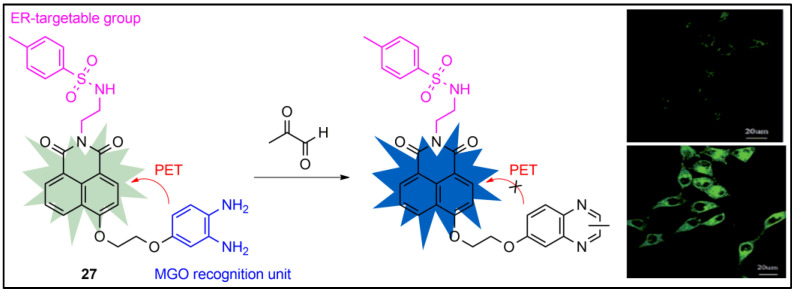
The recognition mechanism of **27** towards MGO. Confocal fluorescence images for monitoring exogenous MGO. HeLa cells were incubated first with **27** (10 mM), then with different concentrations of MGO—0 μM, (control, upper panel) and 50 μM (lower panel) for 2 h (*λ*_EX_ = 405 nm, collected at 420–480 nm). Adapted with permission from [99]. Copyright (2018) The Royal Society of Chemistry.

As a reactive dicarbonyl that is primarily produced by glycolysis in living objects, methylglyoxal (MGO), and its increased levels, is associated with diabetes and related complications. This encouraged Yang et al. to construct a selective probe for methylglyoxal (probe **27**) and examine its chemosensing properties [99]. Probe **27** was designed on PET “*fluorophore*–*spacer*–*receptor*” format using a 1,8-naphthalimide fluorophore and *o*-phenylenediamine recognition unit. Due to the effective PET in molecule **27**, it was non-emissive. However, after exposure to methylglyoxal for 2 min, the PET quenching *o*-phenylenediamine underwent chemical conversion in which the PET was inhibited. As a result, the 1,8-naphthalimde quantum yield increased from 0.008 to 0.244. Furthermore, probe **27** showed good bio-biocompatibility and low cytotoxicity according to MTT to MCF-7 and HeLa cells. It was also applied to the visualization of methylglyoxal in diabetic mice tissues (Figure 26).

**Figure 27:**
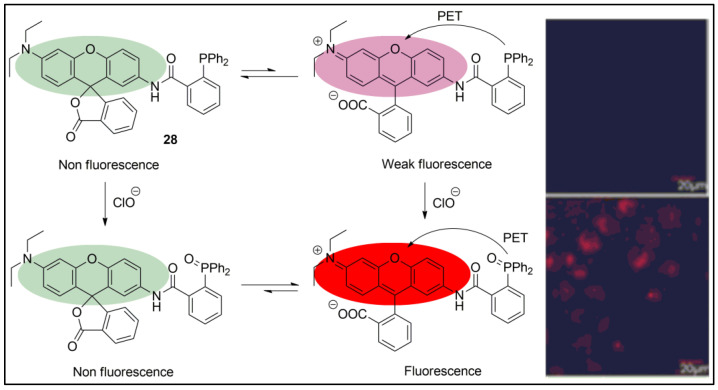
Proposed structural interconversions of probe **28** before and after the addition of ClO^−^. Confocal fluorescence images of living L929 cells incubated with **28** (3 μM) for 1 h (upper panel) and additionally treated with ClO^−^ (9 μM) for 30 min (lower panel). Red channel at 574–638 nm. *λ*_EX_ = 488 nm, Scar bars 20 μm. Adapted with permission from [100]. Copyright (2017) Elsevier.

The great importance of intracellular ROS imaging, which is associated with different diseases, motivated Huang et al. to design fluorescence probe **28** capable of detecting hypochlorite as a typical ROS (Figure 27) [100].

This probe was based on fluorine fluorophore and diphenylphosphinobenzoate recognition moiety using the classical PET “*fluorophore*–*spacer*–*receptor*” model. The presence of diphenylphosphinobenzoate involved a PET process to the fluorine and only a very weak fluorescence was observed. However, after exposure to hypochlorite, the recognition unit in **28** was oxidized, the PET was cut off and strong fluorescence emissions appeared. The probe exhibited cell membrane permeability and was used for imaging of ClO^−^ in living L929 cells.

**Figure 28:**
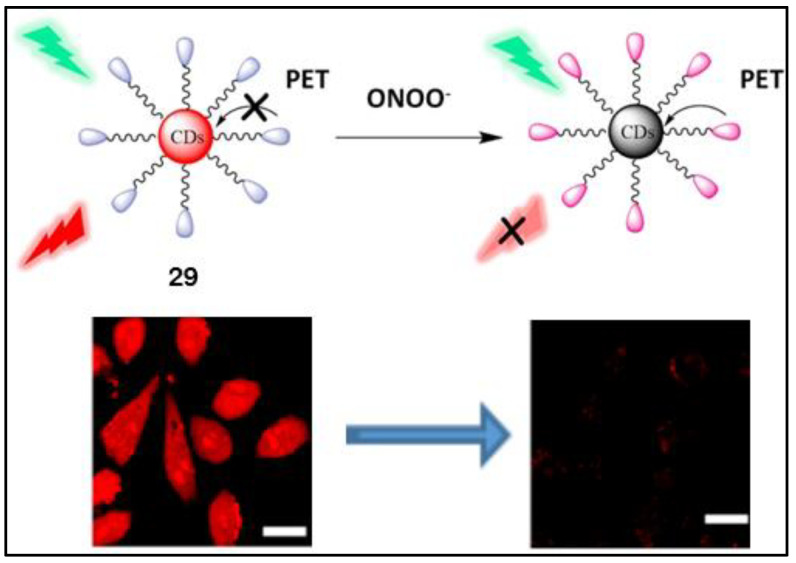
Principle of the ONOO^−^ nanoprobe **29** based on PET. Confocal fluorescence imaging of MCF-7 cells incubated with the nanoprobe **29** (50 μg/mL) and then treated with LPS (1 µg/mL) and IFN-γ (100 ng/mL). The fluorescence emission is collected from 555 to 780 nm. Scale bars 25 µm. Adapted with permission from [101]. Copyright (2017) Elsevier.

As a ROS agent connected to different diseases, peroxynitrite (ONOO^−^) also is an object of intracellular PET fluorescent imaging and observation. For example, the presented in Figure 28 probe **29**, comprising ferrocene functionalized carbon dots, was prepared for the selective determination of peroxynitrite [101]. This nano-system showed a strong red fluorescence centered at 650 nm. In the presence of peroxynitrite, the peripheral ferrocene units were oxidized and a PET process from the periphery to the focal carbon dot occurred. As a result, the former fluorescence was quenched efficiently by 80%. The fluorescence intensity of nanoprobe **29** decreased linearly in the range of 4 nM to 0.12 μM ONOO^−^. The detection limit was found to be 2.9 nM, and the probe was used for the imaging of ONOO^−^ in MCF-7 cells. Moreover, the probe showed high biocompatibility and low cytotoxicity according to an MTT assay against MCF-7 cells (90% cell viability after incubation with 10–50 μg/mL of the probe).

**Figure 29:**
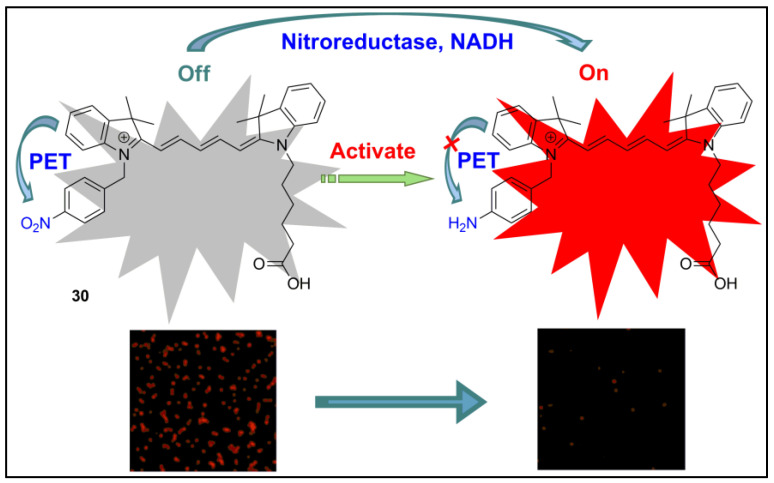
Design strategy of probe **30** for the detection of NTR. Confocal images of Methicillin-resistant *Staphylococcus aureus* (MRSA) incubated with probe **30** (10 μM) in the absence (left image) or presence (right image) of NTR inhibitor (dicoumarin—0.1 mM), *λ*_EX_ =633 nm and *λ*_EM_ =700 ± 50 nm. Adapted with permission from [102]. Copyright (2019) Elsevier.

A common strategy for the utilization of fluorescent probes with high selectivity is based on enzyme-activated fluorogenic probes that “turn-on” the fluorescent signal in the presence of targeted analytes. Based on this approach, probe **30** was successfully implemented in the detection of bacterial nitroreductase, which could be used as a noninvasive tool to diagnose bacterial infections [102]. Compound **30** was non-emissive due to the possible PET from the fluorophore excited state to the electron-poorer nitrobenzyl group, which played the role of a PET receptor unit. After exposure to nitroreductase, the nitro group in recognition moiety was reduced to an electron-rich amine making the PET unfeasible. Owning to the prevented PET, **30** showed intensive fluorescent emissions at about 665 nm (Figure 29). A 10-fold fluorescent increase and detection limit of 36.9 ng/mL was obtained. Meanwhile, bacterial pathogens were also imaged. Low cytotoxicity of the probe was confirmed using MTS assays against HeLa, 293 A, and HepG2.

**Figure 30:**
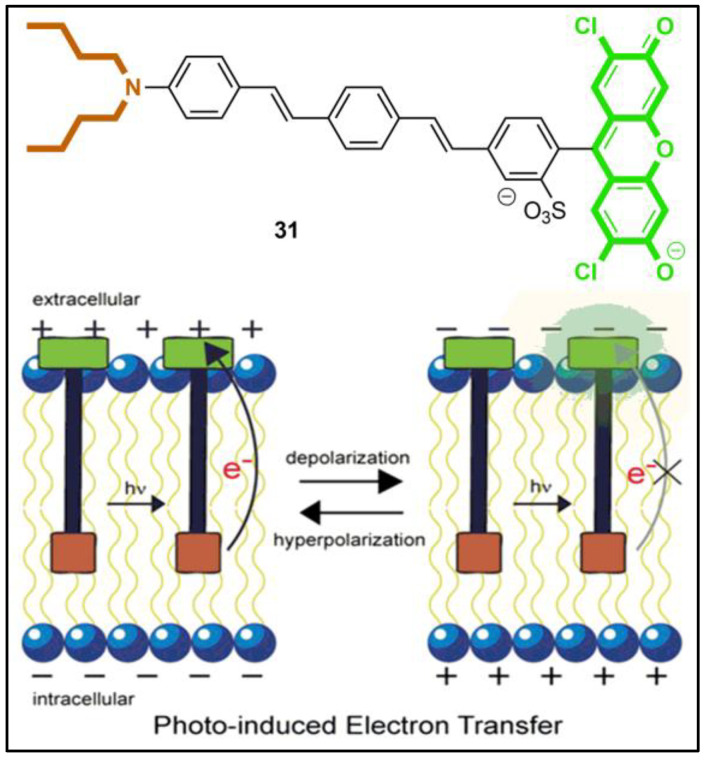
Mechanisms of fluorescent voltage sensing by fluorescent voltage-sensitive dyes (VSDs) [103]. Molecular wire PET **31** depends upon the voltage-sensitive electron transfer from an electron-rich donor (orange) through a membrane-spanning molecular wire (black) to a fluorescent reporter (green). At hyperpolarizing potentials, the electric field is aligned antiparallel to the direction of electron transfer, resulting in efficient PET and quenched fluorescence (**left**). Depolarization aligns the electric field in the direction of PET, decreasing the rate of electron transfer, and increasing fluorescence (**right**).

Recently, a bright idea to apply the PET process in a fluorescent probe **31** for the monitoring of voltage in neurons and neural signals in real time was reported (Figure 30) [103,104].

The nerve signals were changed using rapid potential difference that was set up in a cell membrane by an imbalance of cations such as Ca^2+^, Na^+^, and K^+^ across a lipid bilayer. Miller et al. have integrated a PET fluorophore in the lipid bilayer of the neuron as the fluorophore signaling part stayed outside on the membrane surface while the PET receptor was placed in the membrane. The fluorescent signaling was off due to the PET process that could be modulated from the electric field created by the membrane potential interacting with the electron-donating amino receptor. Thus, the fluorescence intensity of **31** became low or high depending on the accelerated or hindered PET in the membrane (Figure 30). As a result, the changes in neural signals were visualized in real time.

## 5. Fluorescent Probes Based on Excited-State Intramolecular Proton Transfer (ESIPT)

**Figure 31:**
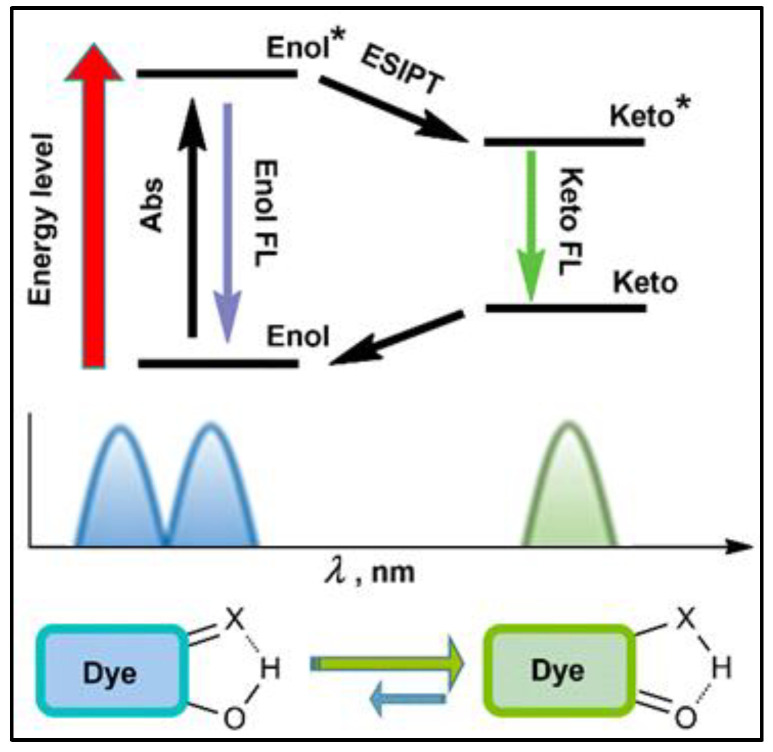
ESIPT photophysics.

The excited-state intramolecular proton transfer (ESIPT) due to increased acidity/basicity in an excited state is a typical phenomenon in molecules, which have a proton (hydrogen)-donating group placed closely to accepting groups [105]. For instance, the hydroxyl hydrogen in salicylic acid decreased its electron density (generated a positive charge) in the excited state while the neighbor carboxylic oxygen increased its electron density (generated a negative charge). This caused a proton translocation from the hydroxyl to the carboxylic group. The general photophysics of ESIPT is represented in Figure 31.

According to the presented photochemical process, the ESIPT fluorophores are characterized by several advantages for sensing purposes, especially in living systems. First, ESIPT probes showed unusually high Stokes shift, which reduced the influence of unwanted self-reabsorption and inner-filter effect. In addition, most ESIPT fluorogenes exhibit dual-emissive signaling owing to the excited state Enol* and the excited state Keto* fluorescence, which allows valuable insights into the bioimaging ratiometric fluorescent measurements.

**Figure 32:**
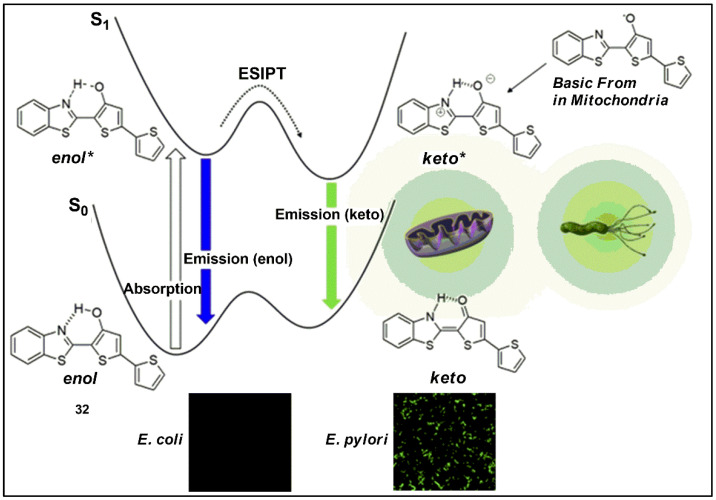
ESIPT probe **32**. Confocal images of *E. coli* and *H. pylori* cells were incubated with 10 μM T_2_(OH)B for 30 min. Adapted with permission from [107]. Copyright (2019) Elsevier.

Because of ESIPT attractiveness, many fluorescent probes for different analytes have been synthesized including physiological pH, biological important cations and anions, ROS agents, and biomolecules [106]. Probe **32** is an example of an ESIPT-based probe for the measurement of pH fluctuations in living organisms [107]. Compound **32** showed blue fluorescence, which rapidly shifted into the green region under slightly basic conditions due to its transition from the enol form to the keto form (Figure 32). This probe was non-toxic and it was used for real-time mitochondrial imaging of Hella cells. In addition, due to the ESIP sensing process, it was applied for the detection of *Helicobacter pylori*. *H. Pylori* can survive under harsh acidic conditions in the stomach due to its urease, which catalyzes the hydrolysis of urea to generate ammonia, which neutralizes the acidic microenvironment around the pathogen. Thus, *Helicobacter pylori* was selectively detected from the slightly pH-responsive probe **32**.

**Figure 33:**
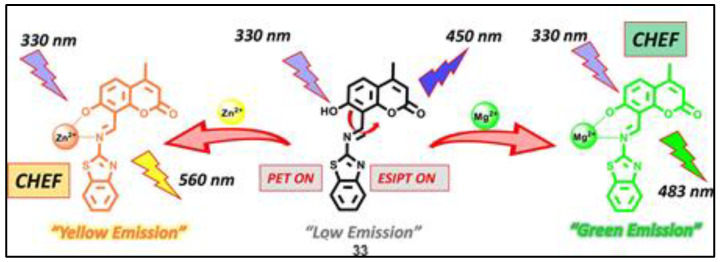
Sensing mechanism of **33** towards Mg^2+^ and Zn^2+^ ions. Adapted with permission from [108]. Copyright (2023) Elsevier.

An interesting example of the ESIPT probe for the detection of biologically relevant cations was that prepared by Singh et al., namely, compound **33** [108]. Probe **33** has a very weak fluorescent emission at 450 nm that was attributed to the possible ESIPT in this molecule (Figure 33). In the presence of Zn^2+^ and Mg^2+^, due to the deprotonation of **33** in the binding process with these cations, the ESIPT was blocked and new bright fluorescent emissions were observed. Moreover, the different cations induced a discriminative fluorescent output. After complexation with Zn^2+^, **33** shed yellow fluorescence (560 nm), while Mg^2+^ binding was indicated by green emissions (483 nm). These differences were explained with the differently arisen chelation-enhanced fluorescence. The abnormal concentrations of Zn^2+^ and Mg^2+^ in the living organisms were related to several diseases including epilepsy, Alzheimer’s disease, cerebral ischemia, and cancer. That is why the intracellular imaging of these ions could be an excellent diagnostic tool. Thus, the chemosensing properties of probe **33** were tested after the incubation of MDA-MB cells with 20 μM of the probe. It was found that **33** showed excellent intracellular response toward the cations Zn^2+^ and Mg^2+^. In addition, the MTT assay indicated very low cytotoxicity.

**Figure 34:**
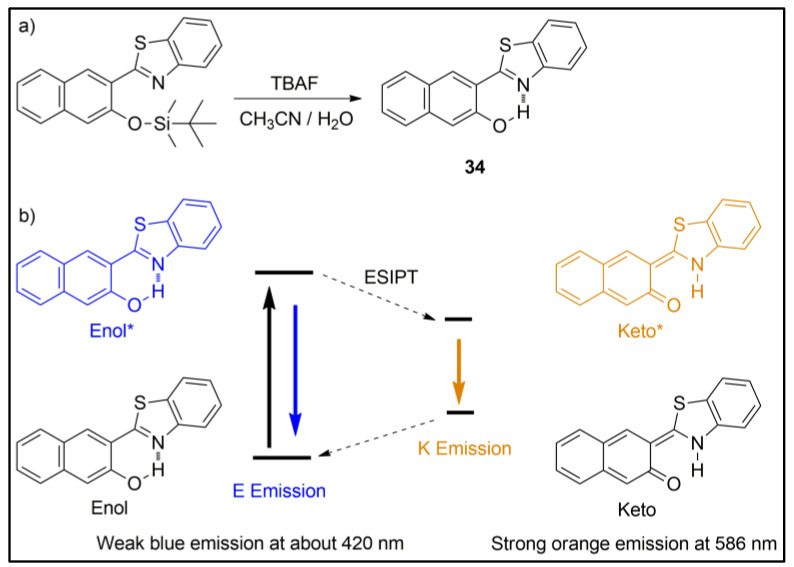
(**a**) Chemical structure of **34** with the equilibrium between protonated and unprotonated species and (**b**) ESIPT process of **34** in the presence of F− [109].

Recently, Mahapatra et al. obtained compound **34**, which was designed as a ratiometric fluorescent probe for the determination of F^−^ anions [109]. It is well known that a higher concentration of fluoride induces apoptosis, causes fluorosis, and leads to nephrotoxic changes and urolithiasis in humans [110,111,112]. Hence, the detection of F^−^ anions plays an important role from a diagnostic point of view. The fluorescent signaling properties of **34** were based on the ESIPT mechanism. The TBS group protected the hydroxyl group of **34**, and due to the lack of proton, the ESIPT process was switched off. As a result, the system showed blue fluorescence with maximum emissions at 415 nm. The introduction of fluoride removed the TBS group through Si-O bond cleavage resulting in a proton-containing hydroxyl fragment. Thus, the ESIPT process was switched on. The obtained ESIPT fluorescence of **34** in the presence of F^−^ was centered at 586 nm (Figure 34). This dual channel response of **34** toward F^−^ was utilized for ratiometric analysis. The detection limit of the probe for fluoride ions was 10.18 μM. The ability of the probe for intracellular F^−^ imaging was also tested. The observed results revealed that the probe was low-toxic against Vero 76 cells, and it could serve as a fluorescent recording of intracellular F^−^.

**Figure 35:**
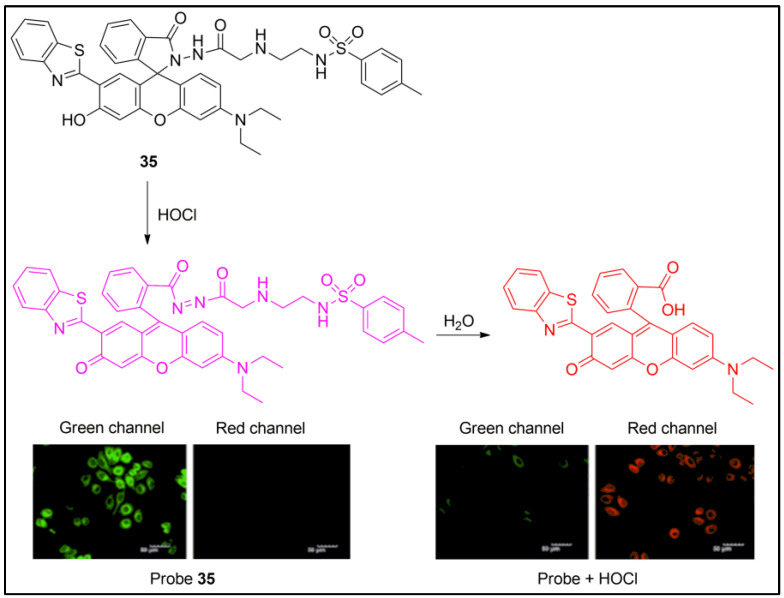
Response mechanism of probe **35** to HOCl. Confocal images of **35** responding to HOCl in HepG2 cells. HepG2 cells were incubated with **35** (10.0 μM) for 15 min prior to incubating with HOCl (20 equiv.) for 15 min. Adapted with permission from [113]. Copyright (2022) Elsevier.

The ESIPT-based probe could be a useful tool for the monitoring of intracellular ROS units. Jiang et al. designed probe **35** as an efficient fluorescent reporter for ClO^−^ in living cells [113]. For the detection of ClO^−^, diacylhydrazine moiety was introduced into rhodamine, which selectively was oxidized into diimide by ClO^−^ and, further, underwent a decomposition in water. This reactive recognition mechanism was confirmed using mass spectroscopic analysis (Figure 35).

The free probe **35** displayed a strong broad fluorescence at 515 nm and a large Stokes shift of 155 nm, which was assigned to the ESIPT process. In the presence of NaOCl, an emission at 585 nm with a small Stokes shift (25 nm) was observed, suggesting the interruption of the ESIPT process. The red-shifted fluorescence was explained by the simultaneous ring-opening reaction in this probe. The fluorescence intensity ratio of emission at 515 nm and 585 nm increased from 0.012 to 5.827, in addition to 0-140 μM NaOCl. The calculated detection limit was 40 nM. Meanwhile, the probe was successfully applied for the detection of exogenous and endogenous HOCl in HepG2 cells (Figure 35). According to the MTT assay against HepG2 cells, **35** showed low cytotoxicity with about 90% viability at 30 μM of the probe.

**Figure 36:**
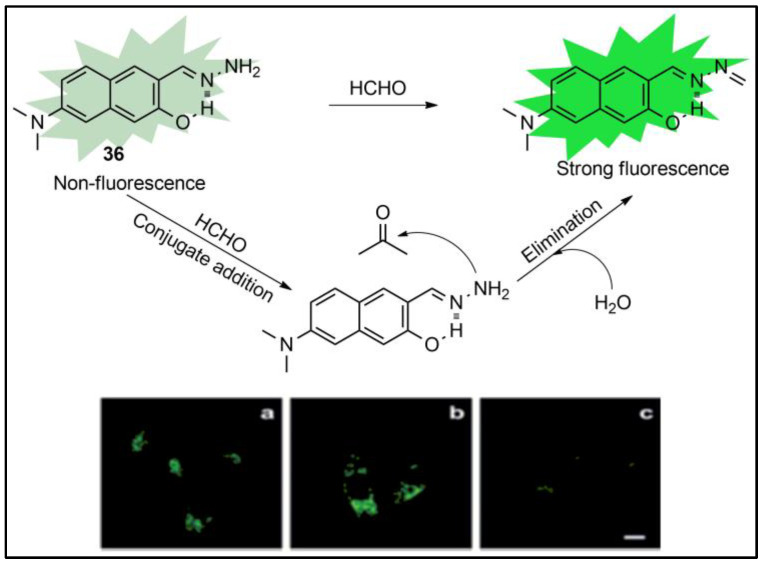
Structure of probe **36** and its mechanism of the reaction to formaldehyde. Confocal microscopy images (**a**–**c**)—endogenous, exogenous, and inhibitory experiments of formaldehyde in HeLa cells. Adapted with permission from [116]. Copyright (2019) The Royal Society of Chemistry.

Formaldehyde is a reactive carbonyl species, which plays an indispensable role in various biological processes. The abnormally high levels of formaldehyde cause damage to biomolecules and cellular dysfunctions and are associated with pathological diseases such as neurodegenerative diseases, diabetes, and even cancer [114,115]. Therefore, currently, significant attention is paid to the detection and imaging of formaldehyde in biological objects. Chen et al. were motivated to prepare selective probe **36** for formaldehyde detection, using a naphthalene core fluorophore and hydrazone responsive unit (Figure 36) [116].

Compound **36** showed low emissive output at 510 nm. However, after a reaction with formaldehyde, the fluorescence intensity was enhanced 14 times due to the ICT-activated fluorescence, after the formation of an electron-withdrawing Schiff base instead of the former electron-donating amine group. This probe was distinguished from other hydrazone-based formaldehyde probes due to its greatly improved selectivity, which was attributed to the presence of ESIPT, which lowered the reactivity of the hydrazone moiety. It was found that **36** exhibited low cytotoxicity (viability over 90% using WTS-1 assay) and was successfully used to image both endogenously generated and exogenously added formaldehyde in Hella cells.

## 6. Fluorescent Probes Based on Fluorescent Resonance Energy Transfer (FRET)

**Figure 37:**
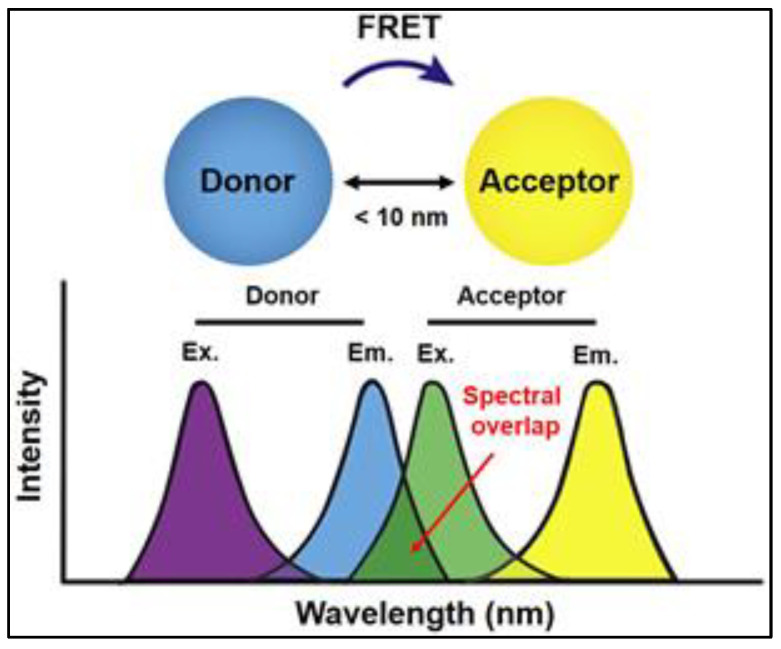
Specific requirements for the detection of FRET signals. The spectra of donor molecule emission (*Em.*) are overlapped with the spectra of acceptor molecule excitation (*Ex.*). The efficiency of FRET appears to be the highest when the energy transferred from *Em.* to *Ex.* is fallen within 10 nm in a parallel orientation.

Fluorescent resonance energy transfer (FRET) is another important photophysical technique that provides a useful fluorescent signal for sensing and imaging application, especially in biological media [18,117,118]. This process occurs by the nonradioactive, resonant transfer of energy from an excited donor fluorophore to a closely placed acceptor molecule in the ground state. It is important to understand that this is not the result of emissions from the donor being absorbed by the acceptor. FRET is observable due to a donor–acceptor dipole–dipole interaction. That is why the general requirement for FRET is the spectral overlap between the fluorescence spectrum of the donor and the absorption spectrum of the acceptor that generates enough energy for the dipole–dipole coupling. Furthermore, it should be pointed out that the FRET process is distance dependent and requires a very close distance (usually 1–10 nm) between the donor and the acceptor. Because of FRET, the excitation of donor fluorophore leads to fluorescent emissions of the acceptor. (Figure 37).

Similar to the ESIPT, the FRET signaling output has some benefits in the construction of fluorescent probes for biomedical applications. First, FRET-based systems offer long communication wavelengths (excitation energy and observed fluorescence signal) resulting in a large pseudo-Stokes shift. Second, on the basis of fluorescence emissions of both the donor and acceptor, ratiometric measurements can be achieved. As was mentioned above, the long-wavelength excitation and the ratiometric signaling reduced the problems of autofluorescence and scattering during fluorescent sensing.

**Figure 38:**
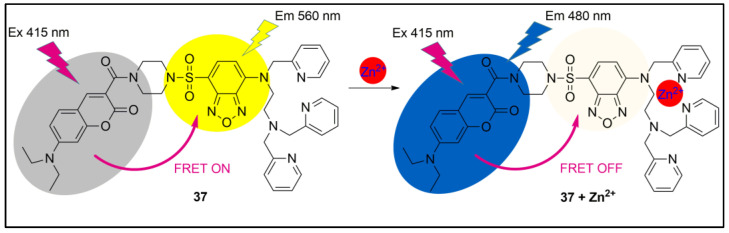
Chemical structure of **37** and schematic illustration of its ratiometric Zn^2+^ sensing mechanism [119].

An interesting FRET-based ratiometric probe **37** for imaging of Zn^2+^ in living organisms was designed by H. Xu et al. [119]. Probe **37** was constructed by the integration of blue emissive coumarin derivatives with an intramolecular charge transfer (ICT) 4-aminooxadiazole fluorophore possessing yellow fluorescence. In order to obtain the selective-sensing properties of Zn^2+^, the 4-aminooxadiazole was functionalized with *N*,*N*,*N′*-tri(pyridin-2-ylmethyl)ethane-1,2-diamine recognition moiety. Due to the effective spectral overlap between the emission of coumarin and 4-aminooxadiazole absorption, a FRET process occurred in **37**, which showed typical energy-acceptor yellow emissions. However, the Zn^2+^ binding decreased the electron-donating ability of the 4-amino group, which weakened the ICT effect in oxadiazole and blue shifted its absorption. Thus, the former spectral overlap was strongly reduced and the FRET was interrupted. As a result, the blue fluorescence of the donor coumarin was observed (Figure 38). Using the fluorescence changes at 480 nm and 560 nm, a ratiometric analysis for the quantitative determination of Zn^2+^ was applied. Probe **37** was cell membrane permeable and showed localization in the endoplasmic reticulum. It showed low toxicity and was used in 3D ratiometric Zn^2+^ imaging of zebrafish larvae’s heads. Based on **37**, new ratiometric protocols for monitoring labile Zn^2+^ homeostasis were realized.

**Figure 39:**
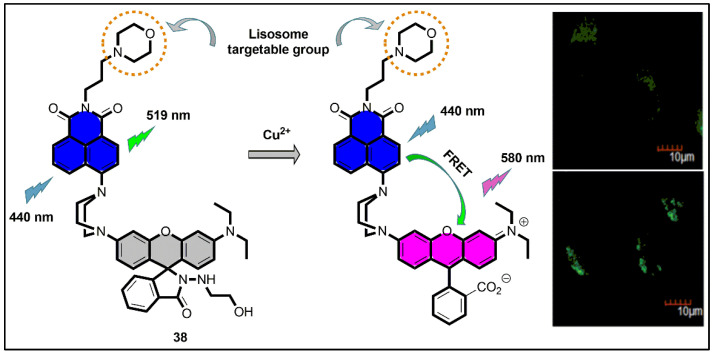
Sensing mechanism of **38** in the presence of Cu^2+^. Fluorescence images of L929 cells with **38** (200 nM) of the green channel (*λ*_EX_ = 559 nm; *λ*_EM_: 581-620 nm) in the absence (upper panel) and presence (lower panel) of Cu^2+^ (1 μM). Adapted with permission from [123]. Copyright (2017) The Royal Society of Chemistry.

Copper plays an important role in many physiological processes and its aberrant homeostasis in lysosomes leads to serious diseases such as Wilson’s disease, Menkes disease, Alzheimer’s disease, kidney failure, and liver diseases [120,121,122]. This motivated Liu et al. to fabricate probe **38**, capable of determining Cu^2+^ fluctuations in the lysosomes [123]. Compound **38** is a bichromophoric system containing yellow-green emissive 1,8-naphthalimide and rhodamine fluorophore. For better distribution in the lysosomes, a morpholine linker was also bonded to **38**. In the absence of Cu^2+^, this probe emitted yellow-green fluorescence from the 1,8-naphthalimide unit. The authors found that the presence of Cu^2+^ induced the hydrolysis of rhodamine from spirolactam to ring-opened xanthene form, which was confirmed by ESI-MS analysis. The rhodamine spirolactam form is colorless and absorbed at the UV region. However, the formed ring-opened xanthene has absorption in the spectral region of the yellow-green fluorescence of the 1,8-naphthalimide unit. This overlap resulted in a FRET process from 1,8-naphthalimide to the rhodamine and a red fluorescence raised together with a fluorescent quenching of the former 1,8-naphthalimide emissions (Figure 39). These changes resulted in a ratiometric response toward Cu^2+^ with a detection limit of 1.45 nM. Due to the morpholine linker, probe **38** was selectively localized in the lysosomes of living L929 cells. The performed imaging of L929 cells demonstrated low toxicity and high sensitivity and selectivity for detection of intracellular Cu^2+^ by probe **38**.

**Figure 40:**
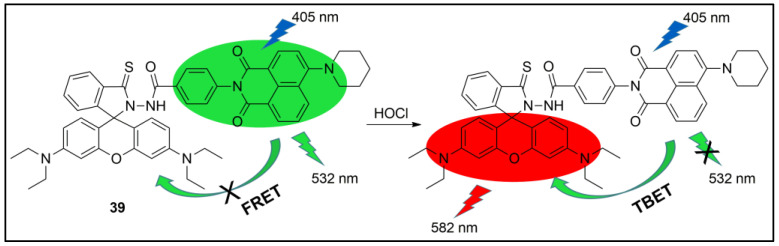
The proposed mechanism of **39** for sensing HOCl [124].

Shen et al. used a similar strategy for the synthesis of the ratiometric probe **39** for the selective determination of HOCl in living cells [124]. In **39**, a spirolactam-closed rhodamine was coupled to a yellow-green emitting 1,8-naphthalimide through a thiohydrazide recognition spacer. The initial yellow-green fluorescence of the 1,8-naphthalimide at 532 nm in the presence of HOCl was red-shifted to 582 nm due to the structural transformation of a rhodamine spirolactam form to ring-opened xanthene, which opened up through-bond energy transfer (TBET) from the 1,8-naphthalimide unit to the rhodamine (Figure 40). It should be noted that after the opening of a rhodamine spirolactam form, FRET is already transformed into TBET. In the case of FRET, a non-conjugated spacer links the donor and acceptor, while at TBET, the donor and acceptor are directly connected via an electronically conjugated bond. The obtained ratiometric signaling revealed a linear response at concentrations of 0–5 µM and a detection limit of 14.5 nM. Meanwhile, **39** was successfully applied to image intracellular HOCl and targeted lysosomes with low cytotoxicity.

**Figure 41:**
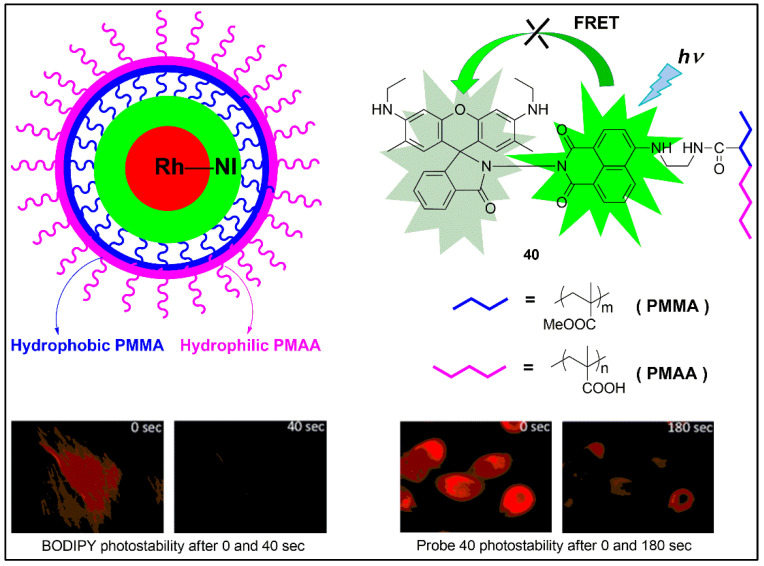
Self-assembly of the FRET-based probe **40** into water-soluble micelles in an aqueous solution. Photostability comparison of the stained HeLa cells by using (left panel) pure BODIPY-labeled actin and (right panel) probe **40** micelles internalized in HeLa cells. Images were taken after different time intervals of continually intense excitation. Adapted with permission from [125]. Copyright (2013) Elsevier.

The higher molecular weight and lower water solubility were the main disadvantages of the FRET-based multi-chromophoric systems, which seriously reduced their biomedical applications. In other to prepare a biocompatible and water-soluble fluorescent probe for ratiometric detection of pH, we have incorporated FRET-based probe **40** into an amphiphilic copolymer, which, in aqueous medium, self-assembles, affording micelles (Figure 41) [125].

The formed micelles served as nano-carriers for the probe and ensured better transport and stability in aqueous media. The pH-sensitive fluorescence response of **40** was based exactly on the same mechanism of probes **38** and **39**. In neutral and alkaline media, the emission of the probe was yellow-green due to the 1,8-naphthalimide fluorescence. In acid media, a rhodamine spirolactam was protonated to a xanthene form that induced FRET from the 1,8-naphthalimide unit, whereby red rhodamine fluorescence appeared. As a result, the fluorescent micelles with the entrapped probe **40** demonstrated a highly pH-sensitive response in water. They are also internalized into HeLa and HEK cells and showed low cytotoxicity and good imaging properties. Moreover, in Hella cells, the fluorescence micelles possessed much better photostability in comparison to the pure organic dye label such as BODIPY.

**Figure 42:**
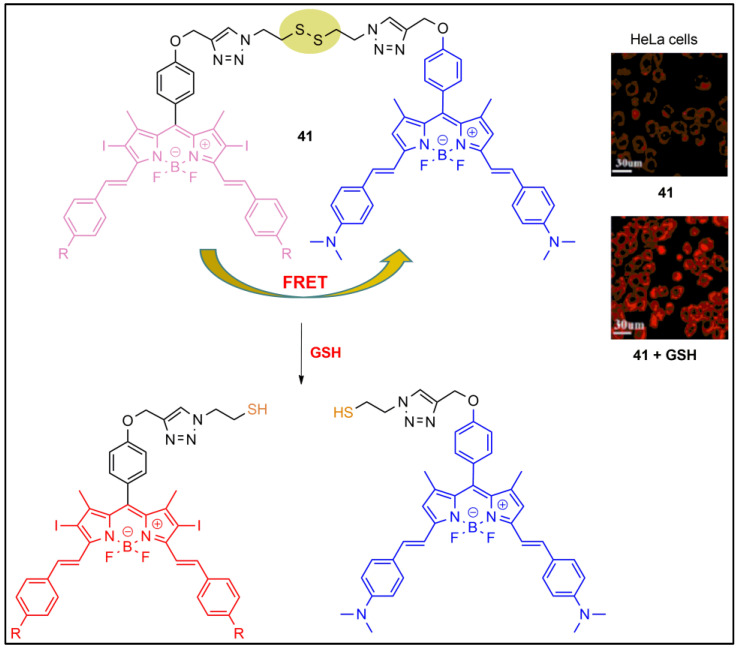
Schematic illustration of the proposed activation mechanism of probe **41**. Confocal images of HeLa cells after incubation with **41** (10 μM) for 8 h (upper image) and cells pre-treated with GSH (5 μM) for 2 h, followed by treatment of **41** (10 μM) for another 8 h (lower image). Adapted with permission from [126]. Copyright (2020) Elsevier.

As we mentioned above, the FRET process is strongly distance dependable. This makes the cleavage reaction another important strategy for the design of FRET-based fluorescence probes. Compound **41** is a typical example of a cleavage reactive probe for the detection of glutathione (GSH), which operates on FRET communication [126]. The monitoring of glutathione in living organisms is very attractive from a diagnostic point of view because its concentration levels in cancer cells are much higher than in normal cells [127]. Probe **41** was designed as a FRET bichromophoric system consisting of three modules, a BODIPY-based energy donating fluorophore, an *N*,*N′*-dimethylamino-based BODIPY energy acceptor, and a bio-reducible disulfide linker (Figure 42).

The disulfide was used as a selective cleavage reactive recognition unit for glutathione. The energy-accepting BODIPY had weak fluorescence emissions and an absorption at 705 nm, which well overlapped with the highly fluorescent emissions of the energy-donating BODIPY. As a result, FRET probe **41** was low-emissive. In the presence of glutathione, the disulfide linker was cut off and both the chromophoric units were separated. Thus, the FRET process was interrupted and the strong fluorescence of the energy-donating BODIPY at 698 nm was emitted. The probe was successfully applied for the imaging of cancer in mice in vivo and in different mice organs ex vivo. Moreover, the released fluorescent BODIPY was an efficient photodynamic agent with negligible dark toxicity. The revealed photodynamic antitumor performance at high glutathione concentrations made this compound an excellent photosensitizer for selective cancer treatment.

## 7. Fluorescent Probes Based on Aggregation-Induced Emission (AIE)

Aggregation-induced emission (AIE) is a relatively new approach for the rational design of fluorescence probes, which rapidly became very popular in the present time [128,129,130]. This phenomenon was observed in 2001 by Tang, who found that some silole derivatives showed high fluorescent emissions in a solid state and were non-emissive in solutions [130]. Usually, traditional organic fluorophores possess bright emissions only in dilute solutions, while their quantum yields of fluorescence are largely weakened in high concentrations due to the aggregation-caused quenching, which results in a non-radiative decay. The opposite results, in contrast with traditional organic fluorophores, inspired the research interest in the development of a new concept for the design of fluorescent materials, particularly with practical applications in the fields of OLED and chemosensing systems. In the past, several mechanistic pathways including J-aggregate formation, conformational planarization, twisted intramolecular charge transfer (TICT), excited-state intramolecular proton transfer (ESIPT), and E/Z isomerization were suggested as the reason for this interesting phenomenon. However, none of them could explain all of the reported AIE systems [131]. AIE compounds played an increasingly important role in the diagnosis and treatment of diseases due to their unique fluorescence properties, biocompatibility, and anti-photobleaching properties [132].

**Figure 43:**
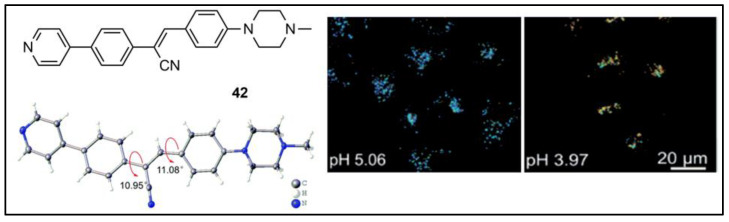
Structure and the single crystal structure of probe **42**. The ratiometric images of the lysosomal pH distribution of HeLa cells without stimulants (left image) and stimulated with 10 mM acetic acid incubated for 8 min at 37 °C (right image). Adapted with permission from [133]. Copyright (2020) The Royal Society of Chemistry.

Compound **42** is an AIE probe that exhibited a 112 nm red shifting of its pH-dependent emission at 503 nm to 615 nm, which is due to the enhanced intramolecular charge transfer after protonation of the pyridine moiety (Figure 43) [133]. Based on both the changes, probe **42** showed ratiometric measurements with highly selective and reversible pH responses. Moreover, this probe exhibited lysosome-targeting ability, good biocompatibility, and excellent photostability. It was successfully applied to map lysosomal pH both in vitro and in vivo. Furthermore, the lysosomal pH changes were monitored during the caudal fin regeneration of a fish model (medaka larvae) using the fluorescent signaling properties of **42**. It was observed that during the regeneration, lysosomal acidification is required to promote autophagic activity for cell debris degradation. These results could be used as a platform for the visualization of various lysosome-involved biological processes such as stress and inflammatory responses.

Lipid droplets are a dynamic organelle that plays a role in some pathological processes, such as fatty liver, neurodegenerative disease, atherosclerosis, and cancer [134,135,136,137].

**Figure 44:**
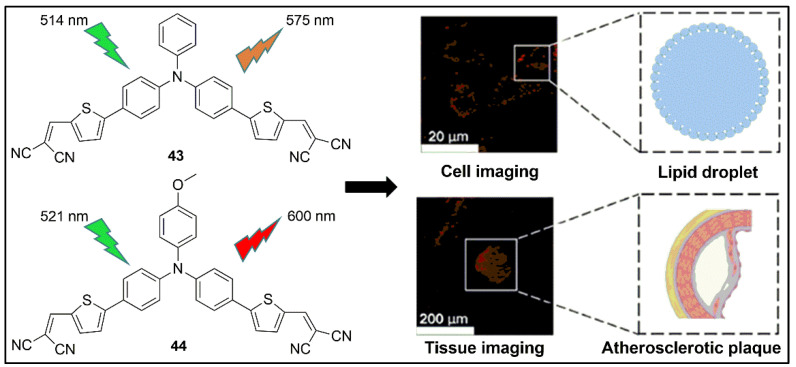
Illustration of **43** and **44** and their LDs-specific cell and atherosclerosis imaging. Adapted with permission from [141]. Copyright (2023) Elsevier.

The AIE probes are widely used for the effective visualization of lipid droplets [138,139,140]. For example, Zhang et al. prepared probes **43** and **44** to monitor lipid droplets for the diagnosis and treatment of atherosclerotic heart diseases (Figure 44) [141]. Both probes (**43** and **44**) were designed on the acceptor–π–donor–π–acceptor model with malononitriles as the electron-acceptor groups, thiophene fragment as a π-bridge, and triphenylamine or 4-methoxy triphenylamine as an electron-donor. Both compounds showed low fluorescence emission in solution or in the aggregated state. However, in sunflower oil, due to the high viscosity of the oil, the molecular motion of these probes was limited, which reinforce AIE fluorescence. Furthermore, they exhibited a large Stokes shift, impressive photophysical properties, great biocompatibility, and excellent photostability. It was found that they showed the great cellular-imaging ability of lipid droplets and were used for the visualization of lipid droplets in Hella cells and mice atherosclerosis models. The obtained results illustrated the great potential of **43** and **44** to provide an option for the diagnosis of atherosclerosis.

Amyloid fibrosis of protein is abnormal self-assembly and fibrillar aggregation of proteins, which causes excessive protein deposition in extracellular tissues and organs and was the origin of Alzheimer’s disease, Parkinson’s disease, and diabetes [142,143,144].

**Figure 45:**
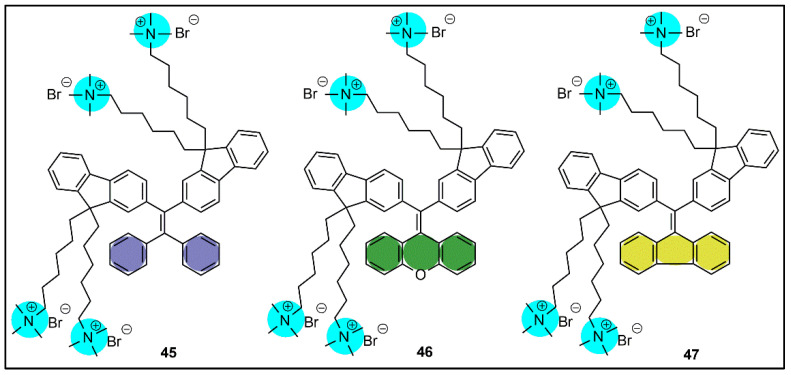
Molecular formulas of probes **45**–**47** [145].

Mei et al. prepared probes **45**–**47** to study amyloid fibrosis [145]. As the main protein model, the authors chose insulin that possessed the determined structure and relatively low molecular weight. Probes **45**–**47** were designed as cationic AIE fluorophores based on different tetraarylethene frameworks (Figure 45).

They exhibited high water solubility and weak fluorescence in solution due to a non-radiative decay. However, they emitted strongly in an aggregated state, which was attributed to the restricted intramolecular motion. Due to the cationic units, the three probes bonded the negatively charged at pH = 9.0 insulin fibrils through electrostatic interaction, which also reduced their intramolecular motion and resulted in a higher fluorescent emission. The detection limits were calculated to be 0.64 nM for **45**, 1.86 nM for **46**, and 5.33 nM for probe **47**. These results showed that compounds **45**–**47** were promising AIE-signaling probes for fluorescence detection of amyloid fibrosis.

**Figure 46:**
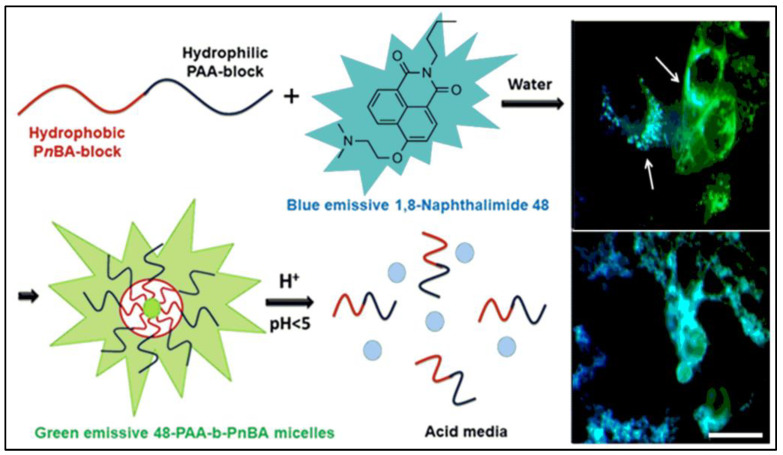
Preparation of pH-sensitive fluorescent **48**-PAA-b-P*n*BA micelles and internalization in living A549 cells visualized by fluorescent microscopy. The cells were incubated for 24 h with fluorescent micelles in different concentrations (0.155 mg/mL—upper image; 0.250 mg/mL—lower image). Bar scale is 50 μm. Adapted with permission from [146]. Copyright (2021) Elsevier.

Recently, we reported a water-soluble nanosized probe as an interesting AIE strategy for intracellular imaging [146]. This probe was obtained after the incorporation of aggregated 1,8-naphthalimide molecules (**48**) in poly(acrylic acid)-*block*-poly(*n*-butyl acrylate) micelles. Owing to the AIE emissions of the core 1,8-naphthalimide units, the micelles exhibited green fluorescence at 560 nm. In acid media (pH < 5), however, the well-defined poly(acrylic acid)-*block*-poly(*n*-butyl acrylate) micelles were destabilized and 1,8-naphthalimides **48** were released as single molecules with strong blue emissions at 450 nm (Figure 46). These fluorescence changes were utilized in a ratiometric fluorescent measurement of pH in water, in a pH window of 3.5-5.5. In addition, the prepared nano-probe showed high cell permeability and low cytotoxicity against living A549 cells, which indicates its high potential for future biomedical applications. Furthermore, it was found that at high concentrations of the micellar probe, most of the cells underwent apoptosis and only monomers were visualized around the apoptotic cell bodies.

## 8. Molecular Logic Gates

The further miniaturization of semiconductors in the electronic field has reached its limit. Therefore, the design and construction of molecular systems capable of performing complex logic functions are now of great scientific interest [147,148,149]. In semiconductor devices, the logic gates work using binary logic, where the signals are encoded as zero and one (low and high current). This process is executable on a molecular level in several ways, but the most common are based on the optical properties of the molecular switches encoding the low and high concentrations of the input guest molecules and the output fluorescent intensities with binary zero and one, respectively. The first proposal to execute logic operations at the molecular level was made in 1988, but the field was not developed until five years later when de Silva [150] experimentally demonstrated the analogy between molecular switches and logic gates. Thanks to their extremely small size, molecular logic devices can penetrate and work in living organisms without endangering their lives. This makes them an integral part of biomechatronics, as they can provide real-time communication between a living object and a machine or device. Molecular logic devices have potential for real-world applications such as object coding, image reproduction, intelligent materials for medical diagnostics, and drug release and activation [151]. Molecular computation is particularly useful for real-time rapid diagnostics in biology and medicine. Such molecular devices can save time for doctors and can ensure safe conditions in epidemics due to analyses performed by the molecular device itself.

**Figure 47:**
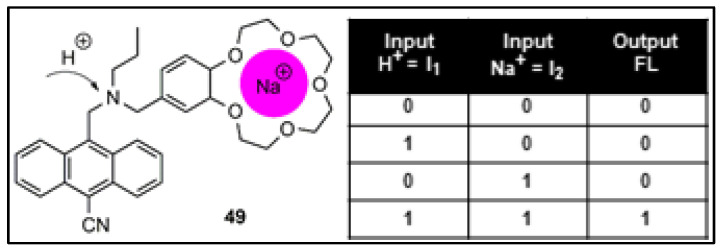
Chemical system **49** performs AND logic operation under the action of two chemical inputs (H^+^ and Na^+^) and the corresponding truth table [150].

The first molecular logic gate was based on anthracene fluorophore **49** that was bounded to two different PET receptors—a tertiary alkylamine for selective recognition of protons and a benzo-15-crown-5-ether for selective recognition of the sodium cation [150]. Due to the two possible PET channels from the receptors, this molecule displayed a strong fluorescence output only when both PET receptors were suppressed. In other words, compound **49** showed high emissive output in the presence of both protons and sodium cations as chemical inputs. This mimics the AND logic gate (Figure 47).

The AND logic gate **49** reported by de Silva could be an interesting tool in medicine for diagnostic and therapeutic applications. Compared to normal tissues, in tumor tissues, the pH is quite acidic and the concentrations of the intracellular sodium ions are significantly higher than in normal tissues. Thus, probe **49**, whose fluorescence was activated in the presence of a high concentration of protons and sodium ions, could serve as a platform for the selective imaging of cancer.

**Figure 48:**
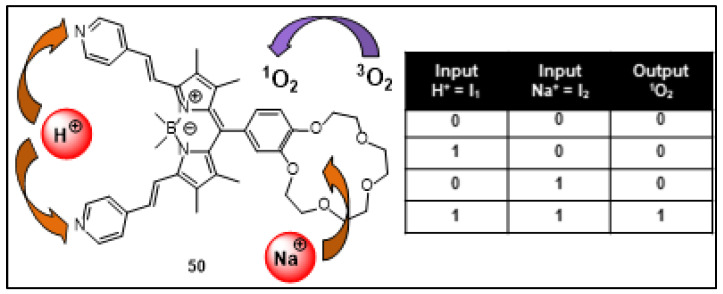
AND logic gate **50** with two inputs (H^+^ and Na^+^) and singlet oxygen as output [152].

Akkaya et al. successfully applied this strategy for the fabrication of a selective anticancer agent in photodynamic therapy **50** (Figure 48) [152]. The AND molecular logic gate **50** was based on the BODIPY photodynamic sensitizer, whose photodynamic activity consists in blocking protons and sodium ions through the pyridine and benzo-15-crown-5-ether receptors bidirectional PET processes. In cancer cells, higher concentrations of proton and sodium ions play the role of chemical inputs, which block both the PET effects, and **50** becomes excellent photodynamic-releasing singlet oxygen as a chemical output. Hence, probe **50** could serve as a platform for the selective photodynamic treatment of cancer cells.

**Figure 49:**
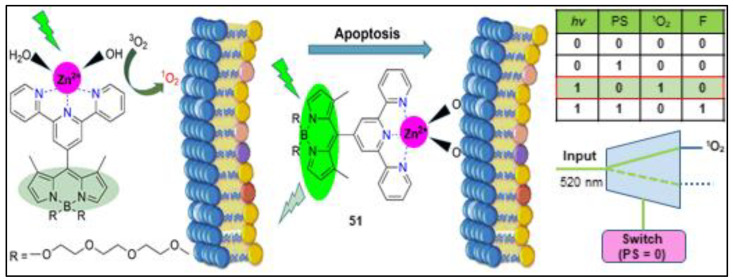
Compound **51** induces apoptosis and then switches to diagnostic mode and fluorescently tags apoptotic cells. Adapted with permission from [153]. Copyright (2018) Springer Nature.

Recently, Akkaya reported another unique molecular logic device as a cancer terminator [153]. The molecular automaton **51** was a photodynamic sensitizer that initially generated singlet oxygen resulting in apoptosis in cancer cells, and then shuts off singlet oxygen generation and produces fluorescent emissions as a result of its interaction with the phosphatidylserines of the changed apoptotic cell membranes. This behavior can be interpreted as a molecular demultiplexer that used light as an input and phosphatidylserines as the switch between the two alternative outputs, which were represented as singlet oxygen and fluorescent emission (Figure 49).

**Figure 50:**
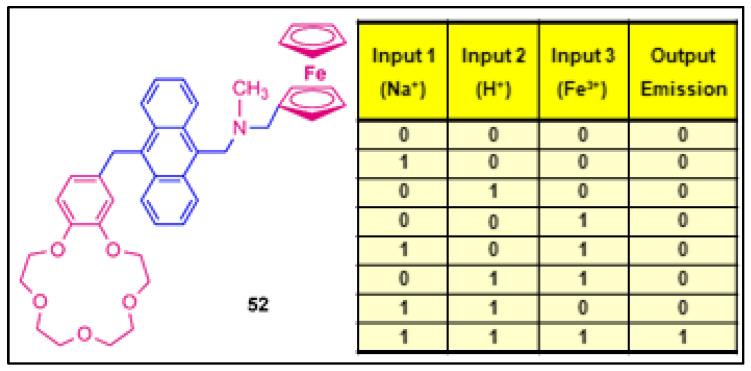
The color-coded design concept of the three-input AND logic gate **52** for Na^+^ (15-crown-5 receptor1), pH (tertiary amine receptor2), and pE (ferrocene redox unit) and the corresponding truth table [154].

Another logic gate with potential as a diagnosing tool was that reported by Magri et al., a three-input AND logic gate **52** [154]. The AND logic gate **52** was based on the receptor_1_–spacer_1_–fluorophore–spacer_2_–receptor_2_–spacer_3_–redox donor format according to PET principles. Similar to probe **49**, it contained tertiary alkilamine receptors and benzo-15-crown-5-ether receptors for the detection of protons and sodium cations. As a third receptor, a ferrocene unit was introduced, whose PET quenching could be interrupted after oxidation by Fe^3+^. Logic gate **52** possesses a high fluorescent output only upon oxidation with Fe^3+^, protonation with methanesulfonic acid, and complexation with sodium ions, simultaneously. This behavior satisfied the three-input AND logic gate (Figure 50). This logic gate represents an improved platform for the detection of cancer in comparison with **49** using sodium ions and protons as inputs because labile redox-active iron has also been attributed to various forms of cancer [155].

**Figure 51:**
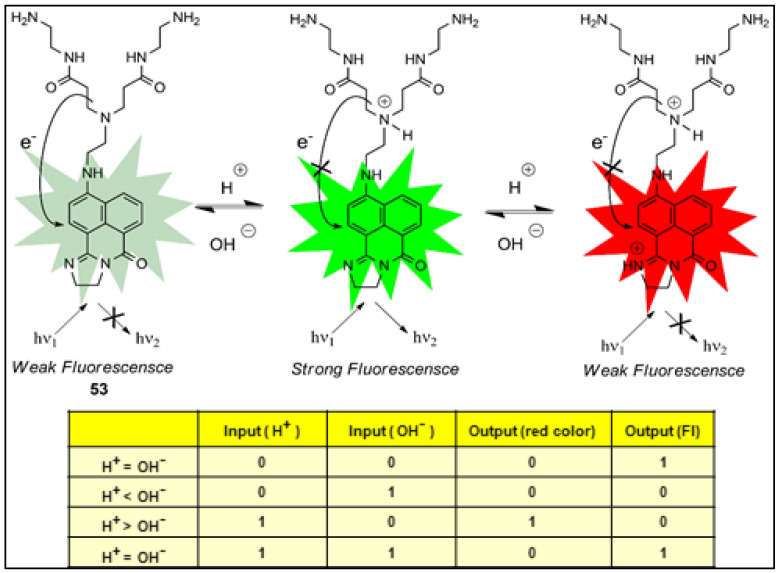
Photophysical behavior of probe **53** as a function of pH and truth table for operation of **53**-based digital comparator [156].

Compound **53** was designed as a water-soluble pH-sensitive probe based simultaneously on PET and ICT [156]. In alkaline solution, probe **53** exhibited a green color and low fluorescence at 520 nm owing to the PET quenching from the tertiary alkylamine receptor fragment. At neutral solution, in pH window 6–7, the PET was blocked and **53** showed bright yellow-green emissions. In acidic solution, protonation of the imine nitrogen (C=N) caused fluorescence quenching again and changed the probe color to red due to enhanced ICT (Figure 51).

Starting from neutral media and using H^+^ and OH^−^ as chemical inputs, the monitoring of fluorescence and red color as outputs gave rise to a magnitude digital comparator based on probe **53**. The magnitude digital comparator is a combinational logic circuit, whose function is to compare two binary numbers. When both inputs were equal (H^+^ = OH^−^), the output fluorescence is one. When adding the acid, i.e., H^+^ > OH^−^, the output red color was one. The addition of base, i.e., H^+^ < OH^−^, resulted in zero for both inputs (Figure 51). Molecular magnitude digital comparators can be a very helpful tool for diagnostics because they show in which direction the abnormal analyte concentrations were placed—the higher concentrations were related to certain diseases, while the lower concentrations were related to others. Moreover, **53** could be of particular interest because it compared pH levels to the physiological pH.

## 9. Fluorescent–Drug Conjugates for Diagnostic and Therapy

Cancer is one of the leading causes of death worldwide. For this reason, major biomedical science efforts are focused on increasing the effectiveness of anticancer therapies. Promising in this direction are two scientific areas under active investigation—advanced chemotherapy and targeted drug delivery. It is only in the last ten years that the idea of combining these within a multifunctional “theranostics” system, combining diagnostic and therapeutic capabilities, has emerged and developed, showing promising results. Theranostic agents are capable of delivering, and subsequently releasing, drug molecules into target tissues and monitoring therapeutic outcomes in real-time. This intelligent form of “all-in-one“ treatment, aims to allow a multifold higher concentration of the imaging agent and/or drug to be achieved in the areas of interest, which strongly influences both the diagnostic potential and the therapeutic effect and ensures the destruction of tumors without affecting healthy tissues and organs, at the right time, with the right dose. Its main advantages over classical methods of diagnosing or treating tumors are the real-time detection of cancer-associated biomarkers, reduction in the side effects of chemotherapeutic agents, due to their poor pharmacokinetic properties and non-specific distribution in the body, and minimizing the likelihood of overtreatment.

Theranostic agents are macromolecular conjugates including a covalently bonded antitumor drug, imaging marker, and targeting component. As targeting agents use high-affinity molecules, such as antibodies, peptides or peptide fragments, aptamers, etc., their role is to target theranostics to specific molecular targets present in cancer cells. The imaging agents are needed to identify and locate the tumor before treatment, provide imaging information about the delivery and distribution of the theranostic platform in the tumor, and obtain feedback on the therapeutic effect after treatment [157]. Among the imaging agents, fluorescent compounds provide promising opportunities to elucidate the drug delivery pathway and intracellular release of the drug molecule due to their non-invasiveness, radiation-free, high sensitivity, and real-time monitoring [23]. The recent advances in the design and synthesis of drug conjugates for fluorescent monitoring in drug delivery and tumor therapy are discussed in several reviews [22,23,24,157,158].

**Figure 52:**
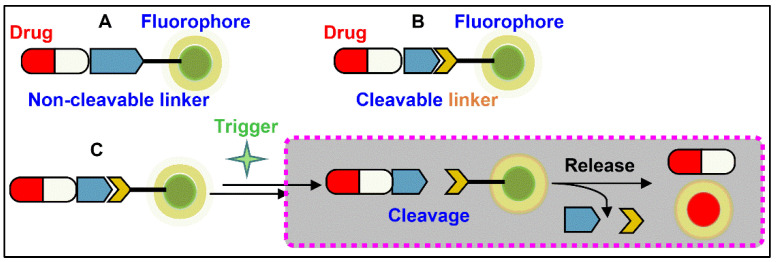
Schematic representation of two main types of fluorophore-drug conjugates: (**A**) non-cleavable conjugates; (**B**) cleavable conjugates; (**C**) Design principle of fluorogenic and release-based cleavable conjugates [24].

In drug-delivery systems, the drug is bound to a fluorescent molecule by means of a non-degradable linkage in a drug-fluorescent dye conjugate (non-cleavable conjugates) or with a biodegradable linker to form cleavable conjugates (Figure 52). Compared to the first group of conjugates, which do not degrade in biological conditions—neither upon transport in the bloodstream nor while being delivered in target or normal tissues, the cleavable conjugates significantly change their spectral properties when the linker is ruptured, and thus the signal drug is released. When the fluorescent dye is bound to the drug, it exists in a non-fluorescent “off” state, whereas when the drug is released, the dye generates a fluorescent signal and switches to a fluorescent “on” form [23].

**Figure 53:**
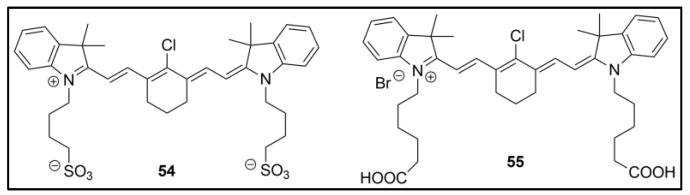
Structure of compounds **54** and **55** [160].

Typical examples of non-cleavable fluorescent-drug delivery systems are heptamethine cyanine dye (HMCD)-drug conjugates [159]. The development of non-invasive and effective technologies for imaging tumors based on HMCDs began more than a decade ago with the work of Yang et al., who reported that the heptamethine cyanine dyes **54** and **55** (Figure 53), with near-infrared fluorescence emission profiles, can detect tumors at metastatic sites and cancer cells in biological fluids, without uptake and retention by normal cells [160].

**Figure 54:**
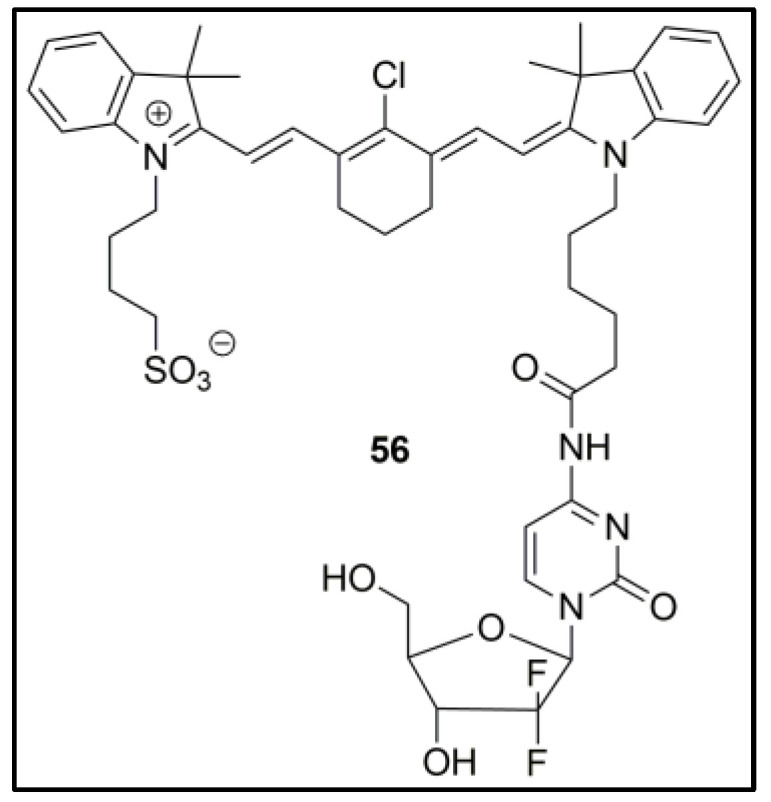
Structure of compounds **56** [161].

In this regard, Wu and colleagues synthesized the conjugate **56** (Figure 54), by covalently linking gemcitabine to the carboxyl group of the dye and explored its potential for the detection and treatment of brain tumor xenografts and prostate tumor metastasized in mouse brain [161].

This conjugation with an appropriate modification of one side chain of the heptamethine carbocyanine dye did not change the electronic properties of the aromatic backbones in order to maintain the fluorescence and tumor-specific targeting properties. On the other hand, the conjugation of gemcitabine at the 4-(*N*)-amino group in the pyrimidine ring via amide bond has emerged as one of the most versatile methods for producing gemcitabine prodrugs without compromising drug activity [162]. The **56** conjugate penetrates the blood–brain barrier and blood–tumor barrier and increases the bioavailability of the drug in the tumor. Ex vivo NIR imaging has confirmed significantly increased retention of the conjugate in mice brains relative to other organs with an increase of up to eightfold in signal intensity. Furthermore, treatment with it has resulted in significantly reduced growth of intracranial U87 tumors in mice.

**Figure 55:**
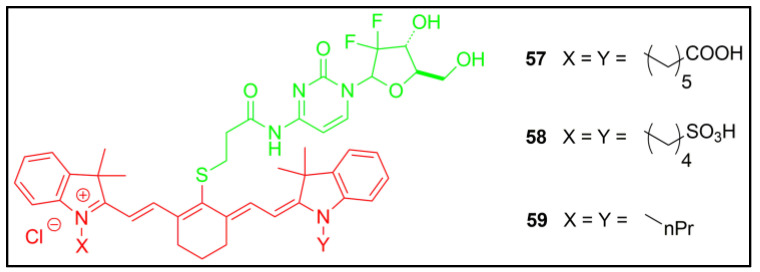
Structure of compounds **57**–**59** [163].

Conjugates **57**–**59** (Figure 55), obtained by the nucleophilic substitution of the meso-Cl atom in the molecule of four HMCDs with gemcitabine, are localized in glioblastoma tumor cells, have absorption maxima in the NIR region, similar to the parent dyes, and show a similar therapeutic effect to gemcitabine but at one-third the molar dose [163]. It is easily metabolized to gemcitabine and a modified HMCD intermediate within 3 h, with an in vivo half-life of only 1 h. This suggests that meso-Cl conjugates are cleared from the tumor faster than their meso-Cl intact counterparts because they cannot form covalent adducts with albumin.

**Figure 56:**
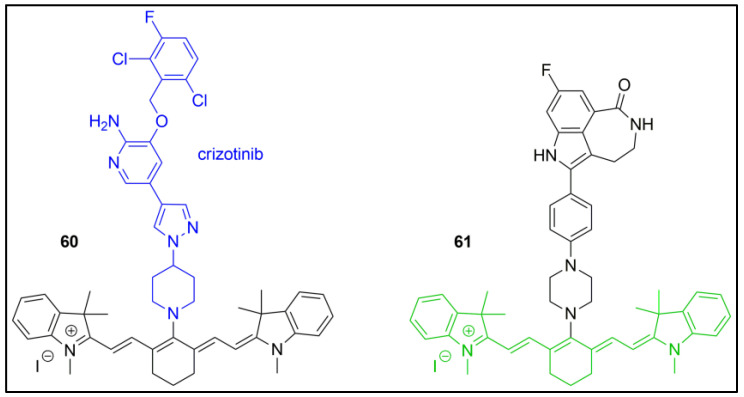
Structure of compounds **60** and **61** [164,165].

The meso-Cl conjugate **60** (Figure 56) showed 100-fold enhanced cytotoxicity over the parent anaplastic lymphoma kinase inhibitor [164]. This improvement is even more pronounced (492-fold) when the above conjugate is combined with temozolomide—a standard drug for the treatment of glioblastoma.

Choi and co-workers [165] synthesized a new drug–dye conjugate **61** (Figure 56), which is a combination of the poly ADP ribose polymerase (PARP) inhibitor rucaparib and heptamethine cyanine dye **60**. The resulting compound demonstrated strong cytotoxic activity with nanomolar potency in three different patient-derived glioblastoma cell lines. The observed potency is at least 780-fold higher than the modified rucaparib. The potency of the conjugate was positively influenced by the addition of temozolomide; although, temozolomide itself did not have any effect in reducing the cell number due to the inherited resistance of these cell lines to temozolomide treatment.

**Figure 57:**
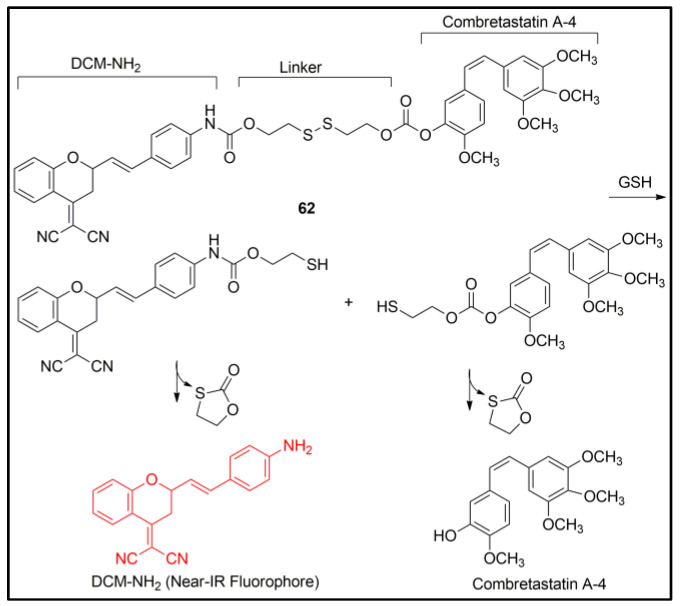
Mechanism of **62** disulfide bond cleavage [166].

Kong Y. co-workers have developed disulfide-based fluorescent-drug conjugate **62** by conjugating on NIR fluorescent molecule DCM-NH_2_, glutathione (GSH)-activatable disulfide linker, and Combretastatin A-4 [166]. The conjugate has no NIR emissions. This is due to the loss of the electron-donating ability of the amine group of DCM-NH_2_ when attached to the linker. However, the disulfide bond in the conjugate is rapidly cleaved by GSH, which is relatively abundant in MDA-MB-231 cancer cells, leading to the release of free Combretastatin A-4, along with DCM-NH_2_ that emitted a NIR fluorescence emission at 650 nm (Figure 57).

**Figure 58:**
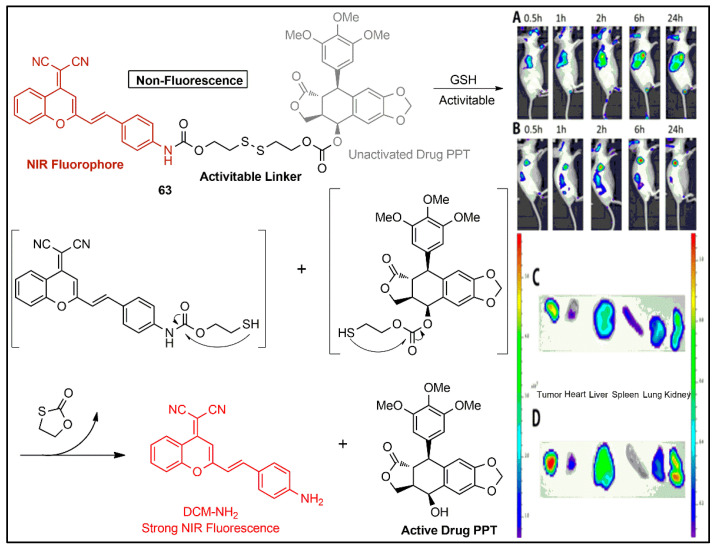
PPT release mechanism of the activatable prodrug by treatment of GSH. In vivo imaging of nude mice bearing tumor after intravenous injection of (**A**) **63** (1 mg/kg PPT) and (**B**) mPEG-DSPE/**63** (1 mg/kg PPT) at various time points (0.5, 1, 2, 6, and 24 h). Ex vivo fluorescence images of the different organs of mice, including kidney, lung, spleen, liver, heart, and tumor, which were sacrificed at 24 h post-injection with (**C**) **63** and (**D**) mPEG-DSPE/**63**. Adapted with permission from [167]. Copyright (2017) American Chemical Society.

The first NIR fluorescence-based prodrug of podophyllotoxin (PPT) **63** is a promising theranostic agent for treating and monitoring the progress of cancer therapy with high efficacy and reduced adverse effects, in which both the NIR fluorescence of dicyanomethylene-4*H*-pyran (DCM-NH_2_) and the cytotoxicity of drug molecule PPT are quenched when covalently connected [167]. The endogenic GSH induces **63** to generate PPT and DCM-NH_2_, resulting in enhanced NIR fluorescence in living cells. Compound **63** showed much lower cytotoxicity to the 293T cells compared to the PPT because of its low concentration of intracellular GSH. By encapsulating **63** in the hydrophobic part of biodegradable copolymer 1,2-distearoyl-sn-glycero-3-phosphoethanolamine-*N*-[methoxy(polyethylene glycol)-2000] (mPEG-DSPE) and the assembled mPEGDSPE/**63** micelles acquired aqueous solubility and tumor-targeting ability (Figure 58).

**Figure 59:**
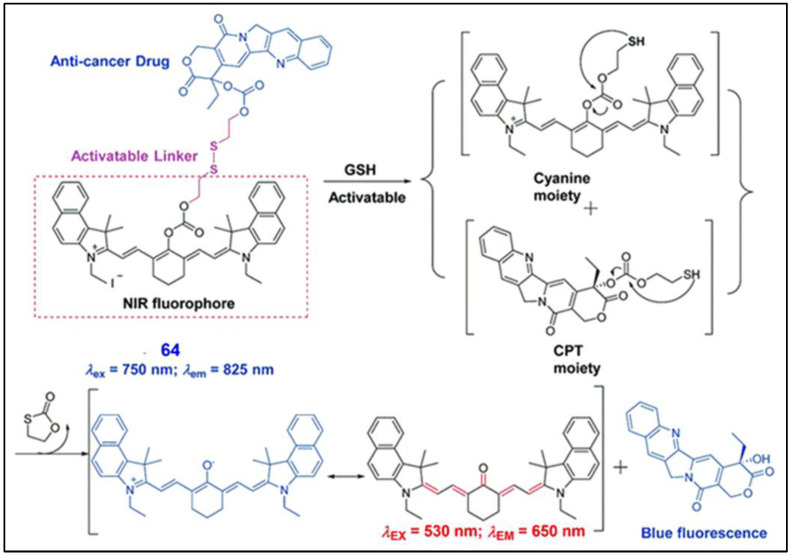
Proposed mechanism in CPT activation and fluorescent variation of the prodrug **64** by the treatment of GSH [168].

**Figure 60:**
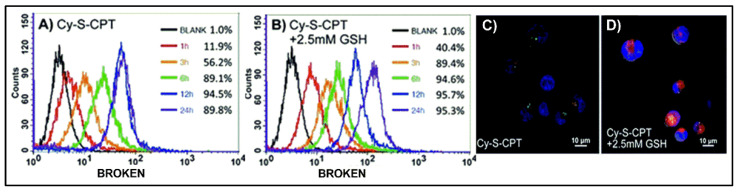
Cellular uptake and intracellular trafficking of prodrugs **64** (Cy-S-CPT) in BCap-37 cells: flow cytometry analysis of the cellular uptake and activation for **64** (**A**,**B**) with and without 2.5 mM GSH, and images of BCap-37 cells cultured with **64** with (**C**) or without (**D**) GSH for 2 h. Red signal indicates the fluorescence from cyanine dye (*λ*_EX_ = 530 nm, *λ*_EM_ = 650 nm) representing the prodrug activation by GSH. Adapted with permission from [168]. Copyright (2016) The Royal Society of Chemistry.

Prodrug **64** of camptothecin (CPT) linked to a cyanine dye via a disulfide bond exhibits a NIR fluorescence emission feature at 825 nm [168]. The cleavage of the disulfide bond in **64** by endogenous GSH activates the CPT and induces a fluorescence shift to 650 nm, thereby providing dual fluorescent channels to real-time track the prodrug biodistribution and activation in vivo (Figure 59). Through this dual-channel NIR fluorescence, bioimaging can overcome the “blind spot” in the metabolic kinetics of the prodrugs in a particular organ or tissue (Figure 60) [168].

Hypoxia, a state of poor oxygen supply to cells (with O_2_ levels <0.1 mmHg), is a common feature of solid tumors, which is associated with disease progression as well as resistance to radiotherapy and chemotherapy, and is one of the main targets in the development of new antitumor drugs and theranostics [169,170].

A reduction in the number of compounds containing azo bonds, nitroaromatic heterocyclics (nitrobenzyl, nitrofuran, imidazole, etc.), *N*-oxides, and quinones by overexpressed enzymes (nitroreductases, oxidoreductases, and flavoproteins) in the tumor hypoxia environment can be used to disclose the active form of a prodrug, hence providing a chemotherapeutic effect [158]. Accordingly, azo bonds, nitroaromatic, nitroheteroaromatic, and quinonic units all can consider hypoxia-sensitive linkers [24,159].

**Figure 61:**
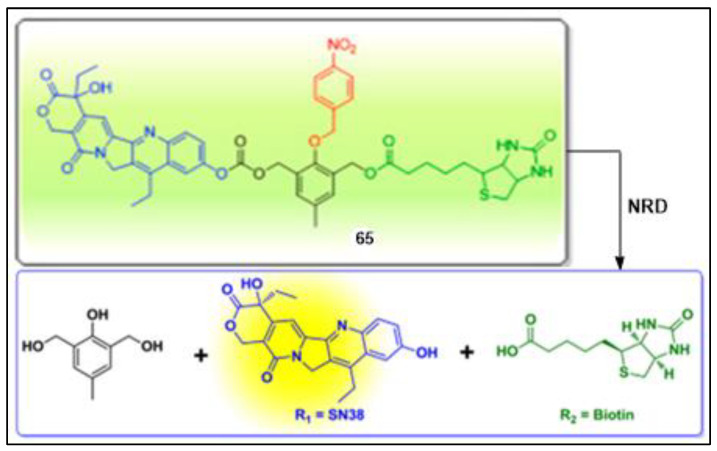
Hypoxia activation of compound **65**. Schematic presentation of drug release in the presence of nitroreductase (NRD) [171].

Kumar R. et al. developed an antitumor theranostic agent **65**, bearing biotin (as a tumor-targeting unit), and the fluorescent anticancer drug SN38, which is activated in hypoxic conditions and showed high therapeutic activity and enhanced fluorescence emission in A549 and HeLa cells and spheroids, which exhibit high expression levels of biotin receptors [171]. The elevated nitroreductase (NRD) activity in the tumor cells under hypoxic conditions served to reduce the nitrobenzyl group in the conjugate to the corresponding aniline derivative, which is inherently unstable and releases the active form of SN38 as a result of an electronic rearrangement (Figure 61).

**Figure 62:**
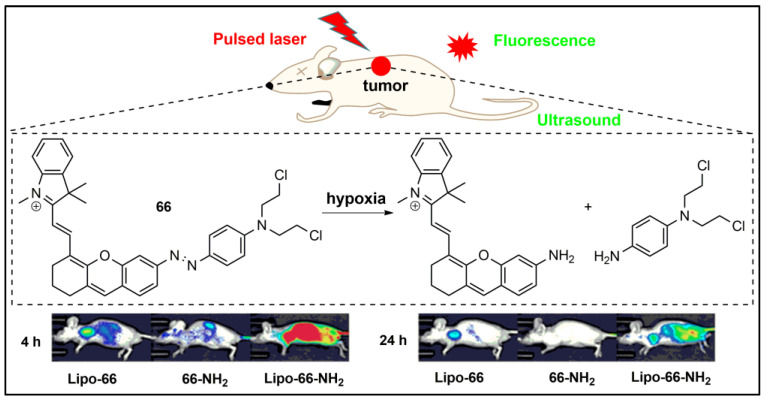
Illustration of the response of **66** toward hypoxia in tumor-bearing mice model. Typical fluorescence images of HepG2 xenograft tumor-bearing mice upon intravenous injection of **66**-NH_2_ (in PBS containing 1 % DMSO), Lipo-**66** (in PBS), and Lipo-**66**-NH_2_ (in PBS) for 4 h and 24 h. Adapted with permission from [172]. Copyright (2019) Ivyspring International Publisher.

In another study, the theranostic **66** embodies a diazo motif as a hypoxia-responsive cleavable group. When the azo linker is cleaved under hypoxia, a chromophore (**66**-NH_2_) is generated with red-shifted absorption and the active drug nitrogen mustard (a classic alkylating agent) is released as well. **66**-NH_2_ shows prominent absorption at around 680 nm and strong fluorescence at about 710 nm, which makes it an ideal contrast agent for detecting and imaging tumor hypoxia both fluorescently and optoacoustically. Molecule **66**, encapsulated into liposomes with particle sizes in the range of 60 ≈ 100 nm, ensures permeability and a retention effect. For example, for the mouse treated with Lipo-**66**-NH_2_, the fluorescence signal appeared in almost the whole body but mainly in the liver and the tumor site, at 24 h post-injection. In contrast, for the mice treated with the molecular fluorophore **66**-NH_2_, the fluorescence mainly resided in the kidneys and was cleared out of the body more quickly (Figure 62) [172].

A distinctive cancer feature is the lower extracellular pH in the cancer tissues (pH ≈ 6.6–5) compared to the extracellular pH in normal tissues (pH ≈ 7.4), and this hallmark is widely used for the development of acid-labile theranostic systems. Typically, these agents are designed to be readily hydrolyzed in acidic tumor environments to furnish active optical signals and release the drug [159].

**Figure 63:**
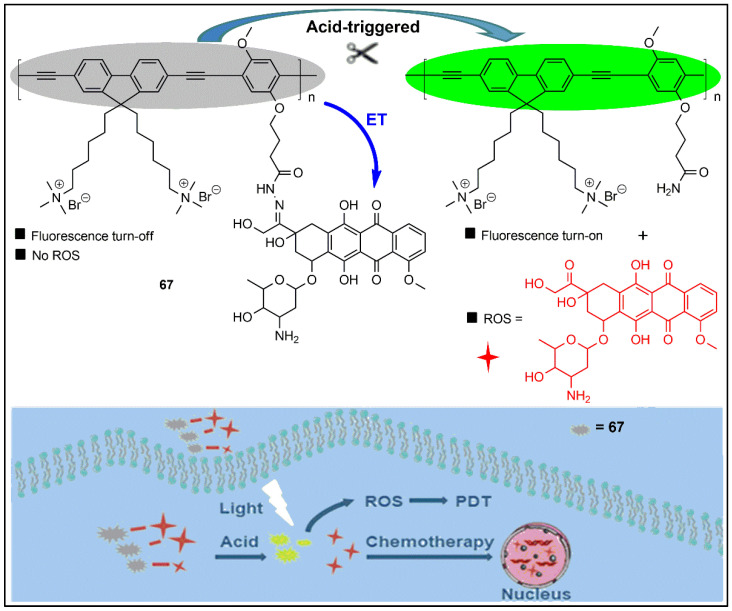
Schematic diagram of pH-responsive drug release and the mechanism of synergistic chemo-/PDT antitumor therapy. Adapted with permission from [173]. Copyright (2023) MDPI.

Recently, Zhang C. and co-workers constructed a pH-responsive drug delivery system **67** based on a conjugated polymer for effective synergistic chemo-/PDT antitumor therapy (Figure 63) [173].

The anticancer hydrophobic drug doxorubicin (DOX), is covalently attached to the side chain of the water-soluble polymer poly(fluorene-co-ethynylene) (PFE) via the acid-sensitive acylhydrazone bond in conjugate **67**. The fluorescence of the conjugated PEE is effectively quenched by the energy/electron transfer (ET) between DOX and the polymer. In addition, the amphiphilic polymer skeleton as the drug delivery vehicle contributes to the uptake of the hydrophobic drug by cancer cells. The acidic microenvironment of cancer cells ensures the effective drug release induced by the breakage of the acylhydrazone bond, which results in the fluorescence recovery of the conjugated polymer, contributing to the monitoring of the drug release process. The cytotoxicity of conjugate **67** against MCF-7 cells under white-light irradiation is significantly higher than that of light-irradiated free DOX at the same concentrations, proving the excellent synergistic chemo-/PDT therapeutic effect of **67**.

**Figure 64:**
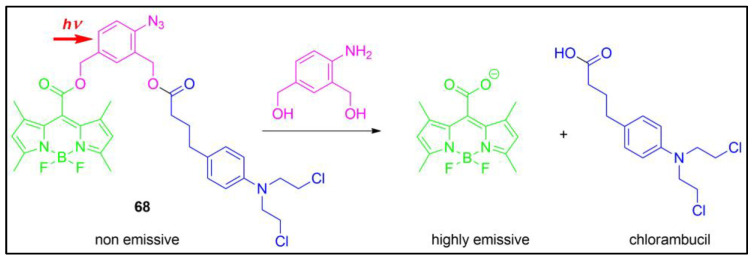
Structure of **68** and release process of drug molecule and fluorescent dye upon light activation [174].

Jang et al. proposed the phototheranostic small molecule **68** (meso-carboxylate-BODIPY—chlorambucil), which is chemically stable under assay conditions [174]. Upon light activation of the conjugate in water, both the drug molecule and the dye are released, which leads to an increase in luminescence by about 1250 times; no toxic nitroso photoproducts products are formed in this process (Figure 64).

Here, we have briefly discussed recent developments of fluorophore–drug conjugates for use in cancer therapy. Fluorescent imaging in theranostics gives new insights into cancer therapy at cellular and molecular levels because it illuminates pathways of drug transport, distribution, and accumulation.

## 10. Conclusions and Perspectives

Currently, significant progress has been made in the development of fluorescent probes for in vitro and in vivo real-time detection and the study of different diseases. Despite the exciting progress in this field, there are still some unresolved challenges to overcome. Most works have carefully examined the toxicity of probes in vitro, but safety assessment on long-term accumulation and toxicity upon in vivo applications is lacking. Furthermore, a lot of the reported probes are insoluble in water and require the use of organic solvents, which increased their toxicity. That is why the design of highly water-soluble probes is very significant for biomedical applications. In diagnostic studies, the analyte-related output signals, dependent on the optical properties of the probes, are vital for the diagnosis of diseases. However, most of the reported fluorescent probes absorb and emit in the visible or NIR I light region. This limits their utility in terms of the in-depth in vivo studying of organ diseases. The development of highly emissive fluorescent probes within the NIR II wavelength region is expected to facilitate the development of systems suitable for the in-depth monitoring of diseases in living organisms. Many fluorescent probes have poor photostability and can be consumed by other active substances. Hence, through fluorescent dye screening and rational design strategy, chemists can cleverly construct functional fluorescent probes with great photostability and chemical stability. In addition, enhancing sensitivity against analytes is another crucial issue to accelerate the progress of clinical application, in order to avoid competition from the cellular microenvironment. The reversibility is another important parameter. Usually, the microenvironments during a pathophysiological process fluctuate with time. The irreversible probe just reflects the microenvironmental changes in one direction, upregulation or downregulation, which cannot fulfill the real dynamic monitoring. Therefore, reaction-based probes are indeed unsuitable for dynamic imaging during a long-term process. It should be pointed out that most fluorescent probes are essentially monochromatic, and such probes are susceptible to interference from external factors. The single-parameter input model of traditional imaging cannot accurately reflect the interactions between multianalyte changes in physiological and pathological-state complicacy and dynamic variation in living organisms. Synchronous imaging for multiple analytes during a certain pathophysiological process can provide cross information on the pathology that is highly desirable. Therefore, the rational design of probes for the synchronous imaging of multiple analytes can be achieved.

In this review, we discussed the main mechanisms and approaches for the rational design of fluorescent probes, finding applications in medical diagnosis and drug delivery systems. In addition, an overview focusing on recent achievements in the development of fluorescence probes and molecular logic devices with potential applications in biology and medicine was presented. We hope that the herein-discussed reports will inspire the researchers working in the field of fluorescence sensing compounds and drug delivery to develop new chemosensing materials with improved practical biomedical applications.

## Data Availability

Not applicable.
